# Prevalence and Trends of Colistin-Resistant *Acinetobacter Baumannii* in the Middle East: A Systematic Review and Meta-Analysis

**DOI:** 10.3390/antibiotics15100987

**Published:** 2026-10-06

**Authors:** Shahd Jadalla, Saddam Akber Abbasi, Usman Abubakar

**Affiliations:** 1 Department of Clinical Pharmacy and Practice, College of Pharmacy, QU Health, Qatar University, P.O. Box 2713 Doha, Qatar; 2 Department of Mathematics and Statistics, College of Arts and Sciences, Qatar University, P.O. Box 2713 Doha, Qatar

**Keywords:** *Acinetobacter baumannii*, colistin resistance, Middle East, systematic review, meta-analysis

## Abstract

**Background/Objectives**: The aim of this study was to synthesize the prevalence and temporal trends of colistin-resistant *A. baumannii* isolates in the Middle East and compare the resistance rates across different countries and clinical specimen types. **Methods**: This systematic review and meta-analysis was conducted in accordance with the PRISMA guidelines. Electronic searches of the PubMed, Scopus and Google Scholar databases were conducted to identify English-language studies that included at least 100 *A. baumannii* isolates. Two independent reviewers screened and assessed the articles and extracted the data from the eligible studies. The data were synthesized using qualitative and quantitative methods. Quantitative synthesis was conducted using R software 4.6.1 with a random-effects binomial generalized linear mixed model (GLMM). **Results**: Eighty-eight eligible studies were included in the analysis. The pooled prevalence of colistin-resistant *A. baumannii* in the Middle East was 1.1% (95% confidence interval [CI]: 0.60–2.00). Lebanon had the lowest prevalence (0.0%), followed by Iran (0.6%; 95% CI: 0.20–1.80), Saudi Arabia (1.5%; 0.30–6.80), and Türkiye (1.6%; 95% CI: 0.60–4.10). The highest pooled prevalence was observed in Egypt (18.6%; 95% CI: 2.80–64.30). The pooled prevalence was 2.5% (95% CI: 0.50–10.90) during 2000–2010, 0.7% (95% CI: 0.40–1.50) during 2011–2019, and 4.0% (95% CI: 0.70–19.3) during 2020–2024, representing an observed 1.6-fold difference between the estimates for 2000–2010 and 2020–2024. Respiratory samples had the highest prevalence of colistin-resistant *A. baumannii* (8.0%; 95% CI: 2.00–26.90), followed by blood samples (3.7%; 95% CI: 1.2–10.40), and urine (1.3%; 95% CI: 0.00–7.23). Samples from wounds/swabs/pus/tissue had the lowest rate of colistin resistance (0.6%; 95% CI: 0.1–6.00). **Conclusions**: The prevalence of colistin-resistant *A. baumannii* in the Middle East remains low, with variation across countries, clinical specimen types, and study periods. Continued surveillance of antimicrobial resistance, strengthening of infection prevention and control measures, and antimicrobial stewardship programs are needed to monitor and address colistin resistance in the region.

## 1. Introduction

Healthcare-associated infections caused by *Acinetobacter baumannii* represent a major global public health challenge due to increasing antimicrobial resistance and limited therapeutic options. This pathogen is a well-recognized cause of ventilator-associated pneumonia, bloodstream infections, urinary tract infections, and wound infections, particularly among critically ill patients in intensive care units, where it is associated with prolonged hospitalization and increased mortality rates [1,2].

*A. baumannii* demonstrates remarkable genomic adaptability, enabling the rapid acquisition of resistance determinants through horizontal gene transfer and chromosomal mutations. This has contributed to the global emergence of multidrug-resistant (MDR), extensively drug-resistant (XDR), and pan drug-resistant (PDR) strains, significantly reducing treatment options and worsening clinical outcomes, including increased mortality and healthcare costs [2,3].

Colistin (polymyxin E) has re-emerged as a last-resort antibiotic for MDR Gram-negative infections, including *A. baumannii*. However, its increased clinical use has been accompanied by a global rise in colistin resistance. Colistin exerts its bactericidal activity by binding to the negatively charged lipid A component of lipopolysaccharide (LPS) in the bacterial outer membrane, disrupting membrane integrity and leading to cell death [4]. Resistance primarily arises through modifications of lipid A that reduce this binding affinity. The most common mechanism involves mutations in the PmrA/PmrB two-component regulatory system, leading to upregulation of phosphoethanolamine transferase enzymes (such as PmrC), which modify lipid A through the addition of phosphoethanolamine. This reduces the net negative charge of the bacterial outer membrane and decreases colistin binding. In addition, loss or structural alteration of LPS due to mutations affecting lipid A biosynthesis pathways has also been described as an important resistance mechanism in *A. baumannii* [4,5].

More recently, plasmid-mediated mobilized colistin resistance (*mcr*) genes have emerged as a significant public health concern. The *mcr* genes encode phosphoethanolamine transferases that mediate the same lipid A modification mechanism but differ by being horizontally transferable between bacterial species. The first plasmid-mediated gene, *mcr-1*, was initially identified in *Escherichia coli* from clinical, animal, and environmental sources, highlighting the potential for zoonotic and ecological spread. Since then, multiple variants (*mcr-1 to mcr-10*) have been identified across diverse bacterial populations, raising concerns about global dissemination of transferable colistin resistance [5]. Although *mcr* genes are less frequently reported in *A. baumannii* compared to Enterobacterales, their emergence underscores the evolving and mobile nature of colistin resistance determinants.

Colistin-resistant *A. baumannii* infections are associated with poor clinical outcomes, including higher mortality rates, particularly among critically ill and mechanically ventilated patients where therapeutic options are extremely limited [1,4,5]. Heteroresistance and undetected resistant subpopulations may also contribute to treatment failure despite in vitro susceptibility, further complicating clinical management [5].

The Middle East region faces a growing burden of antimicrobial resistance due to heterogeneous infection control practices, inconsistent antimicrobial stewardship implementation, and high antibiotic consumption rates. Carbapenem resistance is widely reported across the region, increasing reliance on colistin as one of the few remaining therapeutic options [6,7]. However, variability in reported colistin resistance rates between countries highlights the need for a comprehensive regional synthesis to inform clinical decision-making and policy development.

The COVID-19 pandemic has further intensified antimicrobial resistance pressures due to increased empirical antibiotic prescribing, prolonged hospitalization, and expanded intensive care utilization. These factors may have contributed to the selection and spread of colistin-resistant *A. baumannii*, making comparisons of prevalence across study periods important for understanding resistance dynamics [8].

Therefore, this systematic review and meta-analysis aims to estimate the pooled prevalence of colistin-resistant *A. baumannii* in the Middle East, compare resistance rates across countries, and describe differences in prevalence across study periods, including periods before and after the COVID-19 pandemic.

## 2. Results

### 2.1. Study Screening and Selection

**Figure 1:**
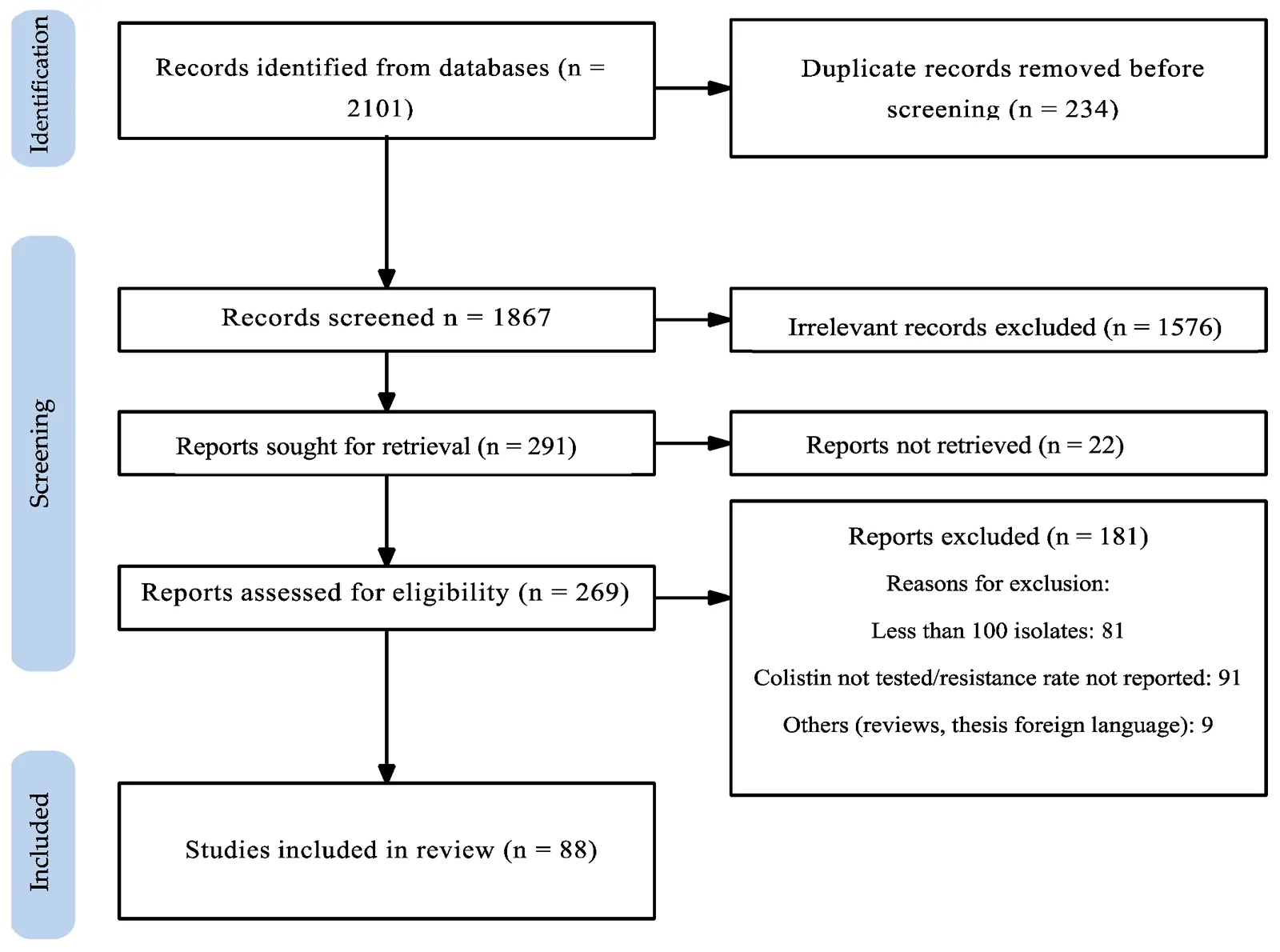
PRISMA flow diagram illustrating the study identification, screening, eligibility assessment, and inclusion process.

The Scopus database search yielded 1453 articles, while PubMed produced 520 articles and Google Scholar generated 128 articles. Overall, 2101 articles were identified from the electronic databases. Initial screening for duplicates identified 234 records, which were subsequently excluded. Eighty-eight eligible studies were included in this systematic review and meta-analysis after full-text assessment. The identification, screening, and selection process is presented in Figure 1.

### 2.2. Study Characteristics

Of the 88 eligible studies included in this review, most (n = 44, 50.0%) were from Iran [9,10,11,12,13,14,15,16,17,18,19,20,21,22,23,24,25,26,27,28,29,30,31,32,33,34,35,36,37,38,39,40,41,42,43,44,45,46,47,48,49,50,51,52], followed by Türkiye (n = 15, 17.1%) [53,54,55,56,57,58,59,60,61,62,63,64,65,66,67], and Saudi Arabia (n = 11, 12.5%) [68,69,70,71,72,73,74,75,76,77,78]. There were five (5) studies from Jordan [79,80,81,82,83], three (3) studies from Lebanon [6,84,85], and two (2) studies each from Egypt [86,87], Iraq [88,89], and Oman [90,91]. The remaining studies were from Kuwait [92], Palestine [93], Qatar [94] and the United Arab Emirates [95].

Single-center studies accounted for more than half of the studies included. The CLSI criteria were the most commonly used criteria for determining colistin resistance. The majority of the studies included isolates cultured from different specimen types, including blood, urine, respiratory samples, wounds, pus, tissue, and swabs. Table 1 summarizes the characteristics of the included studies.

### 2.3. Quality Assessments of Included Studies

**Figure 2:**
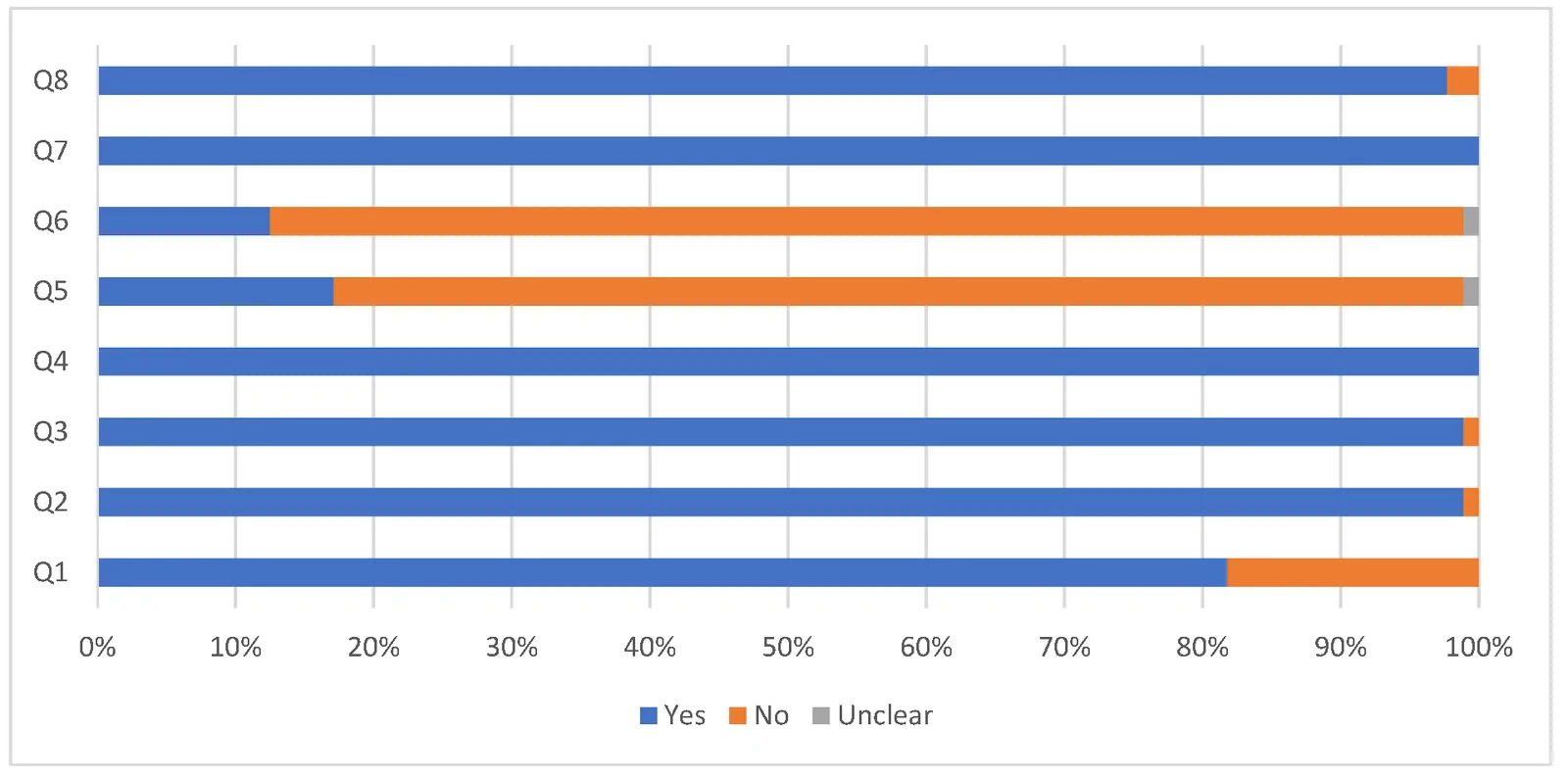
Risk-of-bias assessment of included studies using the JBI checklist. Q1–Q8 denote the eight questions on the Joanna Briggs Institute (JBI) critical appraisal checklist.

At least 80% of the studies included clearly described the criteria for sample inclusion, while 98.9% described the study subjects and setting in detail. Similarly, 98.9% reported exposure using valid and reliable methods, and all included studies used objective, standard criteria for outcome measurement. However, more than 80% of the included studies did not report confounding factors or describe strategies used to address them. All included studies measured outcomes in a valid and reliable way, while 97.7% applied appropriate statistical methods for data analysis. Overall, the studies included were of moderate methodological quality. Figure 2 illustrates the results of the quality assessment.

### 2.4. Prevalence of Colistin Resistance Among A. baumannii Isolates in the Middle East

The prevalence of colistin-resistant *A. baumannii* in the Middle East varied considerably, ranging from 0.0% to 84.2%. Thirty studies (34.1%) reported 100% susceptibility (0.0% colistin resistance). Lebanon had the lowest prevalence of colistin resistance (0.0%) [6,84,85], followed by Palestine (0.7%) [93], the United Arab Emirates (0.9%) [95], Qatar (1.4%) [94], and Kuwait (3.0%) [92]. In Oman, the prevalence of colistin-resistant *A. baumannii* ranged from 1.0% to 8.5% [90,91], while ranges of 0–17%, 0–20.6%, and 0–28% were reported in Saudi Arabia [7,69,70,71,72,73,74,75,76,77,78], Jordan [79,80,81,82,83], and Türkiye [54,55,56,57,58,59,60,61,62,63,64,65,66,67,68], respectively. The prevalence of colistin resistance ranged from 8.0% to 31.7% in Iraq [88,89], 5% to 49% in Egypt [86,87], and 0% to 84.2% in Iran [10,11,12,13,14,15,16,17,18,19,20,21,22,23,24,25,26,27,28,29,30,31,32,33,34,35,36,37,38,39,40,41,42,43,44,45,46,47,48,49,50,51,52,53]. The meta-analysis showed that the overall pooled prevalence of colistin-resistant *A. baumannii* in the Middle East was 1.1% (95% confidence interval [CI]: 0.60–2.00). One study was identified as an outlier [38] and was excluded from the meta-analysis. The pooled prevalence of colistin resistance was lowest in Lebanon (0.0%), followed by Iran (0.6%; 95% CI: 0.20–1.80), Saudi Arabia (1.5%; 0.30–6.80), and Türkiye (1.6%; 95% CI: 0.60–4.10). In Jordan, the prevalence was 1.6% (95% CI: 0.50–8.50), while the highest prevalence was observed in Egypt (18.6%; 95% CI: 2.80–64.30).

### 2.5. Colistin Resistance in A. baumannii Isolates from Different Infection Sites

**Figure 3:**
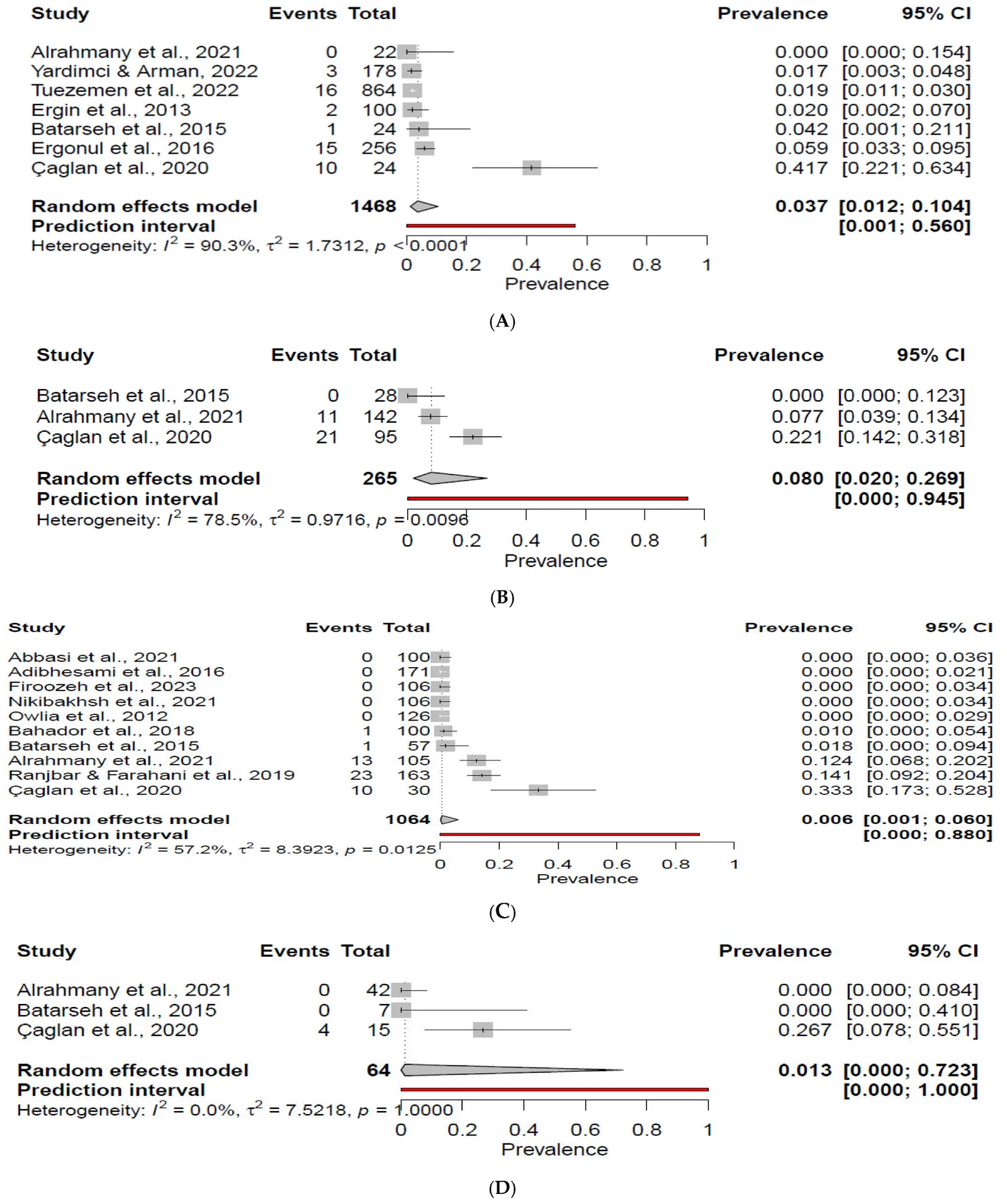
(**A**) Forest plot showing the pooled prevalence of colistin-resistant *A. baumannii* in blood samples [56,61,62,65,67,82,90]. (**B**) Forest plot showing the pooled prevalence of colistin-resistant *A. baumannii* in respiratory samples [56,82,90]. (**C**) Forest plot showing the pooled prevalence of colistin-resistant *A. baumannii* in wound/swab/pus/tissue samples [9,10,13,17,34,35,39,56,82,90]. (**D**) Forest plot showing the pooled prevalence of colistin-resistant *A. baumannii* in urine samples [56,82,90].

Only a few studies exclusively included a single specimen type, while others reported colistin resistance stratified by specimen source. Eleven, seven, four, and three studies provided data for wound/swab/pus/tissue, blood, respiratory, and urine specimens, respectively. Studies that reported 0% prevalence of colistin resistance were included in the meta-analysis. Respiratory samples had the highest prevalence of colistin-resistant *A. baumannii* (8.0%; 95% CI: 2.00–26.90), followed by blood samples (3.7%; 95% CI: 1.2–10.40), and urine (1.3%; 95% CI: 0.00–7.23). Samples from wounds/swabs/pus/tissue had the lowest rate of colistin resistance (0.6%; 95% CI: 0.1–6.00) (Figure 3A–D).

### 2.6. Pooled Prevalence of Colistin Resistance Across Study Periods

**Figure 4:**
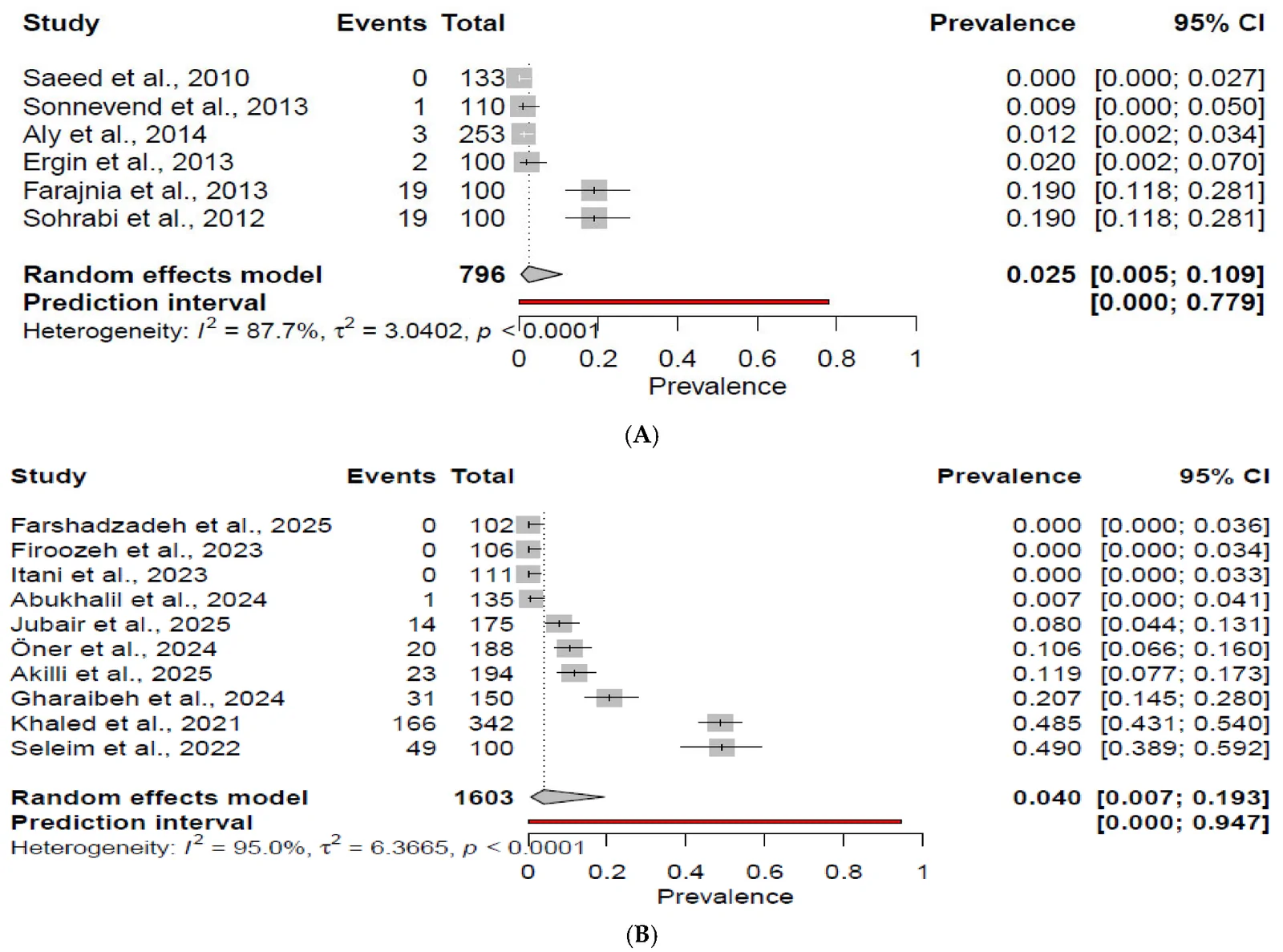

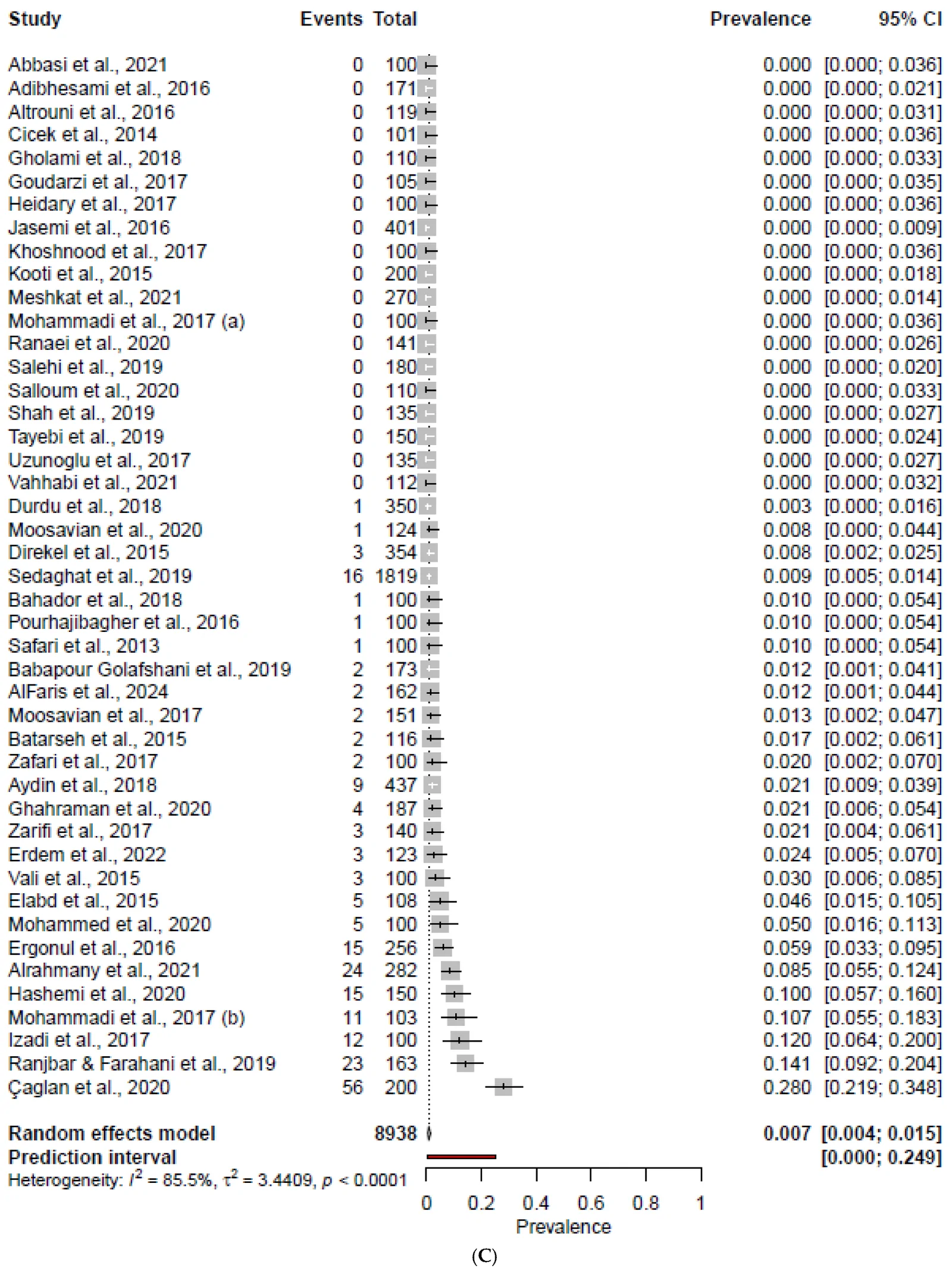
(**A**) Forest plot showing the pooled prevalence of colistin-resistant *A. baumannii* during 2000–2010 [14,47,61,70,76,95]. (**B**) Forest plot showing the pooled prevalence of colistin-resistant *A. baumannii* during 2020–2024 [15,16,53,64,74,83,85,86,89,93]. (**C**) Forest plot showing the pooled prevalence of colistin-resistant *A. baumannii* during 2011–2019 [6,9,10,12,13,18,19,20,21,22,23,24,26,27,28,30,31,32,33,36,38,39,41,43,44,48,49,50,52,54,56,57,58,59,60,62,66,72,77,79,82,84,87,90,92].

Six studies reported the prevalence of colistin resistance among *A. baumannii* isolates collected between 2000 and 2010 [14,47,61,70,76,95], 48 studies included isolates collected between 2011 and 2019, and 11 studies included isolates collected from January 2020 to 2024 [16,17,53,64,74,75,83,85,86,89,93]. Studies that reported 0% prevalence of colistin resistance were included in the meta-analysis. The pooled prevalence of colistin resistance during 2000–2010, 2011–2019 and 2020–2024 was 2.5% (95% CI: 0.50–10.90), 0.7% (95% CI: 0.40–1.50), and 4.0% (95% CI: 0.70–19.3), respectively. The pooled prevalence was 2.5% during 2000–2010 and 4.0% during 2020–2024, representing an observed 1.6-fold difference between the pooled estimates (Figure 4A–C).

## 3. Discussion

This systematic review and meta-analysis evaluated the prevalence and differences in pooled prevalence across study periods of colistin-resistant *A. baumannii* in the Middle East. Studies from 12 of the 17 countries in the region were included, representing 70.6% of Middle Eastern countries, which enhances the regional representativeness of the findings, although evidence gaps remain for several countries. To the best of our knowledge, this is the first systematic review and meta-analysis to comprehensively evaluate both the prevalence and differences across study periods of colistin-resistant *A. baumannii* across the Middle East. The findings provide valuable evidence to support empirical antimicrobial selection, strengthen antimicrobial stewardship programs, and inform infection prevention and control strategies aimed at limiting the emergence and spread of colistin resistance. Overall, the studies included were of moderate methodological quality. Although most studies clearly described the study population, sampling methods, and outcome measurements, confounding factors and strategies to address them were frequently underreported, which should be considered when interpreting the findings. The overall pooled prevalence of colistin resistance in the Middle East was 1.1%, and the prevalence in most countries was below 10%, except for Iraq and Egypt. This pooled estimate is lower than the reported global prevalence of colistin-resistant *A. baumannii* (4%) [96] and the estimated prevalence in the Eastern Mediterranean Region before the COVID-19 pandemic (4%) [97]. In addition, the prevalence observed in the Middle East was lower than those reported in the Region of the Americas (3.2%), Europe (1.8%), and Africa (2.0%) before the COVID-19 pandemic [97]. However, the prevalence is similar to the rate reported in the Western Pacific [97]. These findings highlight the considerable geographic variation in colistin resistance and emphasize the influence of regional differences in antimicrobial stewardship practices, antibiotic consumption, infection prevention measures, and healthcare infrastructure.

Among the included countries, Egypt demonstrated the highest pooled prevalence of colistin resistance. Similar observations have been reported in previous global systematic reviews, in which Egypt consistently ranked among the countries with the highest prevalence of colistin-resistant *A. baumannii* [96]. Differences in reported prevalence between countries and studies may reflect variation in susceptibility testing methods, interpretive criteria (CLSI versus EUCAST), local antimicrobial prescribing patterns, infection prevention practices, and the distribution of clinical specimen types. Previous evidence has shown that studies using broth micro-dilution and EUCAST breakpoints frequently report higher rates of colistin resistance than those using alternative methods [98]. Furthermore, differences in patient populations, healthcare settings, and local epidemiology are also likely to contribute to the observed heterogeneity. Despite the observed variability across countries, the overall findings suggest that colistin retains substantial in vitro activity against *A. baumannii* isolates in the Middle East. Consequently, colistin continues to represent an important therapeutic option for the management of multidrug-resistant *A. baumannii* infections, particularly in settings where alternative treatment options remain limited. This is particularly relevant given the reported resistance of *A. baumannii* to several newer β-lactam/β-lactamase inhibitor combinations, including ceftazidime-avibactam [98], underscoring the continued clinical importance of preserving colistin efficacy through appropriate antimicrobial stewardship.

Previous studies have reported a rising trend in colistin-resistant *A. baumannii* worldwide [96,97]. In the present study, the pooled prevalence of colistin resistance in the Middle East was 2.5% during 2000–2010, 0.7% during 2011–2019, and 4.0% during 2020–2024, representing an observed 1.6-fold difference between the pooled estimates for 2000–2010 and 2020–2024. This observed difference was lower than the change reported in a previous systematic review, in which the prevalence increased from 2% during 2001–2011 to 5% during 2012–2023 [96]. Although causality cannot be established, the higher pooled estimate observed during 2020–2024 may, at least in part, reflect the indirect impact of the COVID-19 pandemic on antimicrobial resistance in the region. This observed difference was also lower than that reported in Western Europe, where the prevalence of colistin-resistant *A. baumannii* increased nearly threefold, from 11% in 2019 to 31% in 2020 [96]. Several factors may have contributed to the higher pooled prevalence observed during 2020–2024. Greater reliance on colistin for the treatment of carbapenem-resistant *A. baumannii* infections may have increased selective pressure for resistance [99]. Furthermore, prior exposure to colistin has been identified as an independent risk factor for infection with colistin-resistant *A. baumannii* [100]. Other recognized risk factors include prolonged intensive care unit (ICU) stay, mechanical ventilation, and the use of invasive medical devices, all of which became more common among patients with severe COVID-19 requiring critical care. Collectively, these factors may have contributed to the emergence and transmission of colistin-resistant strains during the pandemic period. The prevalence of colistin resistance represents a significant challenge for the management of MDR *A. baumannii* infections, particularly because therapeutic alternatives remain limited and resistance to newer agents, including ceftazidime-avibactam and ceftolozane-tazobactam, has also been reported. Consequently, strengthening antimicrobial stewardship programs is essential to optimize colistin prescribing and reduce selective pressure for resistance. In parallel, rigorous infection prevention and control measures, including surveillance, hand hygiene, environmental decontamination, and adherence to isolation precautions, should be reinforced to limit the spread of colistin-resistant *A. baumannii* across healthcare facilities.

Respiratory specimens had the highest pooled prevalence of colistin resistance in the Middle East, in contrast to findings from a global systematic review and meta-analysis, where non-respiratory specimens showed higher resistance than respiratory specimens [96]. This inconsistency may be explained by methodological differences between the two analyses, as the current study stratified resistance by specimen type, whereas the previous study grouped all non-respiratory isolates together. This finding is consistent with the reported association between mechanical ventilation, a key risk factor for ventilator-associated pneumonia, and colistin-resistant *A. baumannii* infections [100]. In contrast, *A. baumannii* isolated from wound, swab, pus, and tissue specimens had the lowest pooled prevalence of resistance compared with respiratory isolates.

The present study has several limitations that should be considered when interpreting the findings. First, only 71% of countries in the Middle East were represented, which may limit the generalizability of the results. Second, restriction to English-language publications introduces potential language bias, as relevant studies published in other languages, including Arabic, may have been missed. Third, 30 of the included studies (34.1%) reported 0% prevalence of colistin resistance. Although these studies were retained in the meta-analysis using a generalized linear mixed model (GLMM), the high proportion of zero-prevalence studies may increase uncertainty around the pooled estimates and affect their precision and interpretation. Fourth, substantial heterogeneity in antimicrobial susceptibility testing methods across studies may have introduced measurement bias and affected the robustness of pooled estimates. Fifth, studies including fewer than 100 isolates were excluded, which may have reduced the inclusion of smaller but potentially relevant datasets. Despite these limitations, this study provides a comprehensive synthesis of the prevalence and temporal trends of colistin-resistant *A. baumannii* in the Middle East.

## 4. Materials and Methods

### 4.1. Study Design

This systematic review and meta-analysis investigated the prevalence and differences in prevalence across study periods of colistin resistance among *A. baumannii* isolates in the Middle East. The study was conducted using the Preferred Reporting Items for Systematic Reviews and Meta-Analyses (PRISMA) 2020 guidelines [101]. The study protocol was registered in PROSPERO (registration number: CRD420261297679).

### 4.2. Eligibility Criteria

#### 4.2.1. Inclusion Criteria

Studies were included if they met the following criteria:

- Studies that reported the prevalence of colistin resistance among *A. baumannii* isolates obtained from human clinical specimens.
- Studies that included isolates collected from countries within the Middle East region.
- Studies that were published in English from database inception to 30 September 2025.

#### 4.2.2. Exclusion Criteria

Studies were excluded if they met any of the following criteria:

- Studies that included fewer than 100 *A. baumannii* isolates for phenotypic susceptibility testing.
- Studies that reported only genotypic resistance without providing phenotypic colistin resistance rates.
- Studies that included environmental or animal isolates without separate, extractable data for human clinical isolates.
- Studies that were previous systematic reviews or meta-analyses, editorials, letters to the editor, or commentaries.
- Studies that were unpublished studies, dissertations, or preprints.

### 4.3. Information Sources

Bibliographic database searches were conducted in PubMed and Scopus to identify eligible studies using predefined keywords (described below). A supplementary search of Google Scholar was performed to identify additional eligible studies.

### 4.4. Search Strategy

The search strategy included the following terms: “prevalence”, “epidemiology”, “colistin”, “polymyxin”, “*Acinetobacter baumannii*”, and “Middle East”, along with the names of individual countries in the region (Bahrain, Cyprus, Egypt, Iran, Iraq, Israel, Jordan, Kuwait, Lebanon, Oman, Palestine, Qatar, Saudi Arabia, Syrian Arab Republic, Türkiye, United Arab Emirates, and Yemen). These terms were combined using Boolean operators (AND and OR) to maximize sensitivity and specificity. In addition, filters for English-language and human studies were applied in both PubMed and Scopus databases to refine the search results.

### 4.5. Selection Process

Articles identified from electronic databases were imported into Endnote^®^ where duplicate articles were identified and removed. Titles and abstracts were then screened to exclude irrelevant studies based on the predefined eligibility criteria. Full-text articles were subsequently assessed for eligibility using the same criteria to determine inclusion in the review. The screening and selection process was conducted independently by one reviewer (UA), while a second reviewer (SJ) independently validated the selection. Any discrepancies between reviewers were resolved through discussion and consensus.

### 4.6. Data Collection Process

Data extraction was performed independently by two reviewers (UA and SJ) using a predefined data extraction form. Initially, UA extracted all relevant data from the included studies, which were subsequently cross-checked and validated by SJ for accuracy and completeness. Any discrepancies in extracted data were resolved through discussion and consensus.

### 4.7. Data Items

Study characteristics and outcome data were extracted from each included study. These included first author’s name, year of publication, country, study setting, study population, study period, criteria used for antimicrobial susceptibility interpretation (CLSI or EUCAST), colistin resistance rate, specimen types, de-duplication of isolates, and antimicrobial susceptibility testing methods.

### 4.8. Study Risk-of-Bias Assessment

Two reviewers (UA and SJ) independently assessed the risk of bias using the Joanna Briggs Institute (JBI) critical appraisal checklist for prevalence studies. Any disagreements were resolved through discussion and consensus. Study quality was categorized as low, moderate, or high risk of bias based on the JBI checklist scoring criteria.

### 4.9. Outcome Assessment and Effect Measures

The primary outcomes of this systematic review and meta-analysis were the pooled prevalence of colistin-resistant *A. baumannii* in the Middle East and country-specific prevalence estimates. Secondary outcomes included the prevalence of colistin resistance stratified by specimen type and differences in pooled prevalence across study periods from 2000 to 2024. For analysis across study periods, studies were grouped into three predefined periods: 2000–2010, 2011–2019, and 2020–2024. The year 2020 was used as the cut-off point to distinguish the study period encompassing the COVID-19 pandemic from the preceding periods.

### 4.10. Data Synthesis Methods

Data were synthesized using both qualitative and quantitative approaches. Study characteristics were summarized using frequencies and percentages, while the prevalence of colistin-resistant *A. baumannii* was descriptively summarized as a range (minimum–maximum values). Quantitative synthesis was conducted using R software version 4.6.1. A random-effects meta-analysis model was applied to estimate the pooled prevalence of colistin resistance in the Middle East and in individual countries that included at least two studies. Approximately one-third of the included studies reported a prevalence of 0%. The analysis was conducted using a random-effects binomial generalized linear mixed model (GLMM). Results were presented using forest plots, and statistical heterogeneity was assessed using the Higgins I^2^ statistic [102].

## 5. Conclusions

The pooled prevalence of colistin resistance among *A. baumannii* isolates in the Middle East was low overall, with variation across countries, clinical specimen types, and study periods. The pooled prevalence was 2.5% during 2000–2010, 0.7% during 2011–2019, and 4.0% during 2020–2024, with an observed 1.6-fold difference between the first and last periods. These findings highlight the need for continued regional surveillance of colistin resistance, alongside strengthened antimicrobial stewardship and infection prevention and control strategies, to support monitoring and management of colistin-resistant *A. baumannii* in the Middle East.

## Figures and Tables

**Table 1:** Characteristics of the included studies.

Author and Year	Country	Study Setting	Study Population	Study Period	No. of Centers	De-Duplication of Isolates	Criteria Used	Colistin Resistance Rate (%)	Specimens Included	Susceptibility Testing Method
Altrouni et al., 2016 [6]	Lebanon	Inpatient and outpatient specimens	119 *A. baumannii* isolates	October 2013 and December 2015	Multi-center	Yes	EUCAST	0/119 (0.0%)	Respiratory tract, skin and soft tissue, urine, blood, and vascular catheters; of note, 19 (13.4%) patients were asymptomatically colonized	Disk diffusion
Abbasi et al., 2021 [9]	Iran	Burn care unit	Patients of all ages (100 clinical isolates)	May 2016 to April 2017	Single center	NA	CLSI	0/100 (0.0%)	Wound swabs	Broth micro-dilution
Adibhesami et al., 2016 [10]	Iran	Burn hospital	Patient of all ages (171 *A. baumannii* clinical isolates from burn hospital (CRAB:168, CSAB:3))	November 2012 to September 2013	Single	Yes	CLSI	0/168 (0.0%) [CRAB Isolates] 0/3 (0.0%) [CSAB Isolates] Overall: 0/171 (0.0%)	Wounds (most)	Disk diffusion and Broth micro-dilution
Aliakbarzade et al., 2014 [11]	Iran	Clinical isolates from hospitalized patients of all ages	103 clinical isolates	NA	Single center	NA	CLSI	19%	Trachea, urine, sputum, blood, catheters, bronchial washing, wounds, abscess drainage, CSF, ascites fluid, pleural effusion	Disk diffusion
Babapour Golafshani et al., 2019 [12]	Iran	Acute burn patients admitted to a tertiary burn hospital	173 acute burn patients	February 2018 to October 2018	Single center	Yes	CLSI	2/173 (1.2%)	Burn wound samples	Disk diffusion
Bahador et al., 2018 [13]	Iran	Burn patients	100 clinical specimens	April–December 2014	Single center	Yes	CLSI	1/100 (1.0%)	Burn wound samples	Broth micro-dilution test
Farajnia et al., 2013 [14]	Iran	ICU (37%) and non-ICU settings	Hospitalized patients of all ages (100 *A. baumannii* isolates)	Between March 2008 and June 2009	Single center	Yes	CLSI	19/100 (19%)	Respiratory specimens (54%), urine (21%), blood (7%), catheters (6%), and other sources (12%)	Kirby–Bauer disk diffusion method
Farajnia et al., 2022 [15]	Iran	ICU	Hospitalized ICU patients of all ages (127 *A. baumannii* isolates)	January 2016 to October 2020	Bi-centric	Yes	CLSI	5/127 (3.9%)	Trachea, urine, blood, wound swabs, fluid (pleural, cerebrospinal), bronchial secretions, catheters, and throat	Agar dilution MIC and E-test methods
Farshadzadeh et al., 2025 [16]	Iran	Teaching hospitals	Clinical isolates from hospitalized patients (102 *A. baumannii* isolates)	January to December 2020	Multi-center	NA	CLSI	0/102 (0%)	NA	Automated broth micro-dilution technique
Firoozeh et al., 2023 [17]	Iran	Burn hospital	Hospitalized burn patients (106 *A. baumannii* isolates)	February 2020 to March 2021	Single center	Yes	CLSI	0/106 (0%)	Burn wound samples only	Broth micro-dilution method
Ghahraman et al., 2020 [18]	Iran	ICU, 2 university-affiliated hospitals	Hospitalized patients in the ICU (187 *A. baumannii* isolates)	July 2017 to May 2018	Bi-centric	Yes	CLSI	4/187 (2.1%%)	BAL: 71.6% (n = 127), wounds: 9.1% (n = 17), CSF: 9.1% (n = 17), blood: 4.3% (n = 8), and urine: 8.66% (n = 18)	Broth micro-dilution method
Gholami et al., 2018 [19]	Iran	Hospital (All units)	Hospitalized patients (110 *A. baumannii* isolates)	November 2016 to July 2017	Bi-centric	Yes	CLSI	0/110 (0.0%)	Wound samples (n = 51, 46.36%), tracheal tubes (n = 37, 33.63)	Broth micro-dilution method
Goudarzi et al., 2017 [20]	Iran	ICU	Hospitalized ICU patients of all ages (105 *A. baumannii* isolates)	Between October 2015 and April 2016	Multi enter	Yes	CLSI	0/105 (0%)	Wounds (n = 65, 61.9%), blood (n = 25, 23.8%), catheters (n = 8, 7.6%), and cerebrospinal fluid (n = 7, 6.7%)	E-test strips
Hashemi et al., 2020 [21]	Iran	Burn hospital	Hospitalized burn patients (150 clinical samples)	2016 to 2019	Single center	Yes	CLSI	15/150 (10%)	Catheters: 13 (8.6%), sputum: 28 (18.6%), trachea: 55 (36.6%), pleural fluid: 10 (6.6%), wounds: 4 (20%) and urine: 40 (26.6%)	Kirby–Bauer disk diffusion
Heidary et al., 2017 [22]	Iran	University teaching hospital	Hospitalized patients of all ages (100 *A. baumannii* strains)	Between January 2014 and May 2015	Single center	NA	CLSI	0/100 (0%)	NA	Kirby–Bauer disk diffusion
Izadi et al., 2017 [23]	Iran	All units	Hospitalized patients of all ages	Between September 2014 and October 2015	Multi-center	NA	CLSI	12/100 (12%)	Sputum (60%), abdominal abscesses (19%), blood (6%), urine (5%), wounds (2%), synovial fluid (2%)	Kirby–Bauer disk diffusion method
Jasemi et al., 2016 [24]	Iran	ICU (52.5%) and non-ICU settings	Inpatient and outpatient isolates (401 *A. baumannii* isolates)	August 2011 to December 2013	Multi-center	Yes	CLSI	0/401 (0.0%)	Respiratory specimens (39.4%), wounds (25%), blood (11%), various body fluids (79%), catheters (27%), urine (2%) and cerebrospinal fluid (1%)	Disk diffusion
Karami et al., 2021 [25]	Iran	ICU and non-ICU settings (predominantly ICU (85.2%), plus burn, trauma/emergency, general surgery and internal wards)	Hospitalized adult patients (108 *A. baumannii* isolates)	NA	Multi-center	Yes	CLSI	45/108 (41.7%)	Tracheal aspirates (81 isolates, 75%), urine (17 isolates, 15.74%), blood (7 isolates, 6.48%), wounds (2 isolates, 1.85%) and cerebrospinal fluid (1 isolates, 0.92%)	Kirby–Bauer disc diffusion
Khoshnood et al., 2017 [26]	Iran	Burn hospital	Burn patients of all ages (100 strains of *A. baumannii*)	June 2013 to August 2014	Single center	NA	CLSI	0/100 (0%)	Urine (12%), sputum (3%), blood (20%), the catheter (25%), and wounds (40%)	Disk diffusion method
Kooti et al., 2015 [27]	Iran	ICU (71.5%) and non-ICU settings	Hospitalized patients of all ages (200 *A. baumannii* isolates)	December 2013 to May 2013	Multi-center	NA	CLSI	0/200 (0%)	Sputum (40.5%), endotracheal tubes (21.5%), wounds (11%), urine (8%), blood (6%), body fluids (6%), others (2%)	Disk diffusion method
Meshkat et al., 2021 [28]	Iran	ICU (76%) and non-ICU wards (burn unit, surgical, internal medicine, emergency)	270 *A. baumannii* isolates from hospitalized patients of all ages	Jan 2014 to Dec 2015	Multi-center	Yes	CLSI	0/270 (0%)	Respiratory discharges (53%), bloodstream (19%), wounds (20%), urine (6%) and cerebrospinal fluid (2%)	Micro-dilution method
Mohajeri et al., 2013 [29]	Iran	ICU and non-ICU settings	Clinical isolates from patients (104 clinical isolates of *A. baumannii*)	November 2010	Multi-center	NA	CLSI	11/104 (10.6%)	Sputum (n = 69), blood (n = 32) and urine (n = 3)	Kirby–Bauer method of disk diffusion
Mohammadi et al., 2017 [30]	Iran	Burn unit	Hospitalized burn patients	August 2013 to March 2014	Single center	NA	CLSI	0/100 (0%)	Burn wounds	Micro-dilution broth method
Mohammadi et al., 2017 [31]	Iran	Hospital-based: burn unit and ventilated patient wards	Hospitalized burn patients and mechanically ventilated patients (103 *A. baumannii* isolates)	April 2015 to March 2016	Bi-centric	NA	CLSI	11/103 (10.7%)	33 burn samples and 70 isolates from ventilated patients	E-test
Moosavian et al., 2020 [32]	Iran	Patients admitted into diverse wards (74.2% from the ICU)	Patient of all ages admitted to two hospitals (124 *A. baumannii* isolates)	July 2011 to January 2013	Bi-centric	Yes	CLSI	1/124 (0.8%)	Tracheal aspirates: 57.3%, CSF: 11.3%, wounds: 10.5%, urine: 8.1%, blood: 3.2%, pleura: 1.6%, catheters: 1.6%, eyes: 0.8%	Kirby–Bauer disk diffusion
Moosavian et al., 2017 [33]	Iran	Two university-affiliated hospitals (all units)	Hospitalized patients from whom clinical Acinetobacter isolates were obtained	December 2014 to October 2015	Bi-centric	Yes	CLSI	2/151 (1.3%)	Tracheal aspirates: 88 (58.3%), blood: 26 (17.1%), wounds: 19 (12.6%), urine: 5 (3.3%), CSF: 5 (3.3%), abscesses: 4 (2.7%), pleural fluid: 4 (2.7%)	Kirby–Bauer disk diffusion
Nikibakhsh et al., 2021 [34]	Iran	Hospital, burn unit (s) ICU and non-ICU settings	Hospitalized burn patients	Between December 2019 and November 2020	Single center	Yes	CLSI	0/106 (0%)	Burn wounds only	Broth micro-dilution method
Owlia et al., 2012 [35]	Iran	Burn unit	Burn patients hospitalized for at least 1 week (126 *A. baumannii* isolates)	August 2010 to February 2011	Single center	NA	CLSI	0/126 (0%)	Burn wound swabs only	Disk diffusion
Pourhajibagher et al., 2016 [36]	Iran	Burn hospital	100 *A. baumannii* isolates from hospitalized burn patients	October 2012 to June 2013	Single center	Yes	CLSI	1/100 (1%)	NA	Broth micro-dilution method
Rahimzadeh et al., 2024 [37]	Iran	ICU and non-ICU settings (including PICU, emergency, surgery, pediatric)	Hospitalized patients with MDR *A. baumannii*	Between 2022 and 2023	Multi-center	NA	CLSI	96/114 (84.21%)	Wounds: (48; 42.1%), sputum: (36; 31.5%), blood: (19; 16.6%), urine (8; 7.0%), catheters (2; 1.7%), CSF (1; 0.8%)	Broth macro-dilution
Ranaei et al., 2020 [38]	Iran	Educational hospitals—inpatient and outpatient settings	Patients of all ages (141 *A. baumannii* isolates)	January to September 2018	Multi-center	Yes	CLSI	0/141 (0.0%)	ETT culture (50.4%; n = 71), blood culture (39.7%; n = 56), urine culture (2.8%; n = 4), wound culture (2.1%; n = 3) and body fluids (cerebrospinal fluid, peritoneal fluid, pleural fluid, vaginal discharge and gastric juice) (5.7%; n = 8)	Broth micro-dilution method
Ranjbar and Farahani et al., 2019 [39]	Iran	Major hospital centers including both ICU and non-ICU settings	Hospitalized burn patients with burn wound infections	January 2016 to July 2018	Multi-center	Yes	CLSI	23/163 (14.1%)	Wounds only	Broth microdilution method
Rostamani et al., 2024 [40]	Iran	Hospital, various specimen types (unit/ward not specified)	120 *A. baumannii clinical* isolates	From April 2021 to July 2022	NA	NA	CLSI	27/120 (22.5%)	Blood: 28 (23.3%), wounds: 22 (18.3%), urine: 36 (30%), and sputum and secretions collected from chest tubes (chest catheters): 34 (28.4%)	Micro broth dilution
Safari et al., 2013 [41]	Iran	ICU	Hospitalized patients in the ICU (100 *A. baumannii isolates)*	June 2011 to October 2012	Multi-center	NA	CLSI	1/100 (1%)	74 tracheal aspirates, 16 blood, 5 urine, 4 sputum and 1 wound sample	Kirby–Bauer disc diffusion method
Salehi et al., 2018 [42]	Iran	ICU (75%), and other wards: surgery, neonatal, orthopedic, neurology, ID and internal medicine	Hospitalized patients of all ages with nosocomial infections (125 clinical isolates of *A. baumannii*)	NA	Single center	Yes	CLSI	0/125 (0.0%)	Sputum (52.8%, n = 66), wounds (12.7%, n =16), urine (12.7%, n =16), blood (9.5%, n = 12), catheters (7.1%, n = 9), eyes (0.8%, n = 1), CSF (2.4%, n =3) and other body fluids (1.6%, n =2)	E test strips
Salehi et al., 2019 [43]	Iran	Hospitalized patients including ICU (n = 134, 74.5%) and non-ICU (n = 46, 25.5%) settings	Hospitalized patients with nosocomial infections due to CRAB	Between August 2016 and February 2017	Bi-centric	Yes	CLSI	0/180 (0%)	Sputum (n = 103, 572%), other sources: not fully specified	Epsilometer test strips (E-test strips)
Sedaghat et al., 2019 [44]	Iran	Burn wards and burn ICU	Hospitalized burn patients	2012–2014	Single center	NA	CLSI	16/1819 (0.9%)	Blood, wounds and others	Disk diffusion
Sepahvand et al., 2016 [45]	Iran	ICU	100 *A. baumannii* isolates from hospitalized ICU patients	NA	Single center	Yes	CLSI	6/100 (6%)	Wounds/ulcers: 60%, respiratory secretions: 20%, urine: 10%, blood: 5%, others: 5%	Modified Kirby–Bauer method + E-test
Shoja et al., 2013 [46]	Iran	ICU	Tracheal tube discharge of hospitalized patients in intensive care units (206 *A. baumannii* isolates)	March 2010 to November 2012	Multi-center	NA	CLSI	0/206 (0%)	Tracheal tube discharge only	Disk diffusion method
Sohrabi et al., 2012 [47]	Iran	ICU (37%) and non-ICU settings	Hospitalized patients (100 *A. baumannii* isolates)	Between March 2008 and June 2009	Single center	Yes	CLSI	19/100 (19%)	Tracheal aspirates (37%), urine (21%), sputum (9%), blood (7%), bronchial washing (6%), catheters (6%), wounds (5%), abscess drainage (3%), cerebrospinal fluid (2%), pleural effusion (2%), and ascitic fluid (2%)	Disk diffusion
Tayebi et al., 2019 [48]	Iran	ICU wards	Patients of all ages admitted into the ICU (150 *A. baumannii* clinical isolates)	February 2016 and July 2018	Multi-center	NA	CLSI	0/150 (0.0%)	NA	Kirby–Bauer disk diffusion
Vahhabi et al., 2021 [49]	Iran	ICU (86.6%) and non-ICU settings	Hospitalized patients of all ages (112 *A. baumannii* isolates)	October 2018 to October 2019	Single center	NA	CLSI	0/112 (0.0%)	Endotracheal aspirates (n = 32), wounds (n = 38), blood (n = 22), urine (n = 12), broncho-alveolar lavage (n = 5) and IV catheters (n = 3)	Broth dilution method
Zarifi et al., 2017 [50]	Iran	ICU	Hospitalized patients (140 *A. baumannii* isolates)	December 2014 to March 2015	Single center	NA	CLSI	3/140 (2.1%)	Tracheal aspirates–71 (50.7%), urine—50 (35.7%), wounds—10 (7.2%), blood culture—9 (6.4%)	Modified Kirby–Bauer disk diffusion method
Zendegani and Dolatabadi, 2020 [51]	Iran	Hospitals	Hospitalized patients with nosocomial infections (100 *A. baumannii* clinical isolates)	NA	Multi-center	NA	CLSI	2/100 (2%)	Urine, blood, skin, and wounds as well as from respiratory tract infections	Broth (macro-broth) dilution
Zafari et al., 2017 [52]	Iran	ICU and non-ICU settings	100 CRAB isolates cultured from hospitalized patients	September 2015 to June 2016	Single center	NA	CLSI	2/100 (2%)	Tracheal aspirates: 44; blood: 38; urine: 9; pulmonary secretions: 4; wounds: 1; catheters: 1	Broth micro-dilution
Akilli et al., 2025 [53]	Türkiye	University hospital including inpatient and outpatient specimens	194 carbapenem-resistant *A. baumannii* isolates from clinical specimens	December 2020 to 2022	Single	Yes	EUCAST	23/194 (11.8%)	Blood, vascular catheters, pleura, cerebrospinal fluid (CSF), wounds, tissue, abscesses, peritoneal fluid, urine, and respiratory samples	Broth micro-dilution
Aydin et al., 2018 [54]	Türkiye	Hospitalized patients with healthcare-associated infections (HAI)	437 *A. baumannii* isolates	1 January 2014 to 31 December 2015	Multi-center	Yes	CLSI	9/437 (2.1%)	Blood cultures (HA-GNBSIs)	Automated system (VITEK 2, Phoenix) automated system (VITEK 2)
Cetin et al., 2022 [55]	Türkiye	Neonatal ICU, infections and skin colonization	103 *A. baumannii* isolates from infections and skin colonization	2018 and 2021	Single center	NA	NA	0/103 (0.0%)	Blood: 61/103 (58.3%), tracheal aspirates: 15/103 (13.6%), urine: 3/103 (2.9%), pleural/peritoneal/CSF samples: 3/103 (2.9%), wounds: 1/103 (1%), skin swabs: 22/103 (21.4%)	Vitek 2
Çağlan et al., 2020 [56]	Türkiye	Hospital-wide clinical samples	200 of *A. baumannii* collected from clinical samples	June 2016 and January 2017	Single center	Yes	EUCAST	Overall colistin resistance: 56/200 (28.0%) By specimen source: Respiratory samples: 21/95 (22.1%), blood: 10/24 (41.7%), pus: 10/30 (33.3%), urine: 4/15 (26.7%), other sterile body fluids: 11/36 (30.6%)	Respiratory specimens (broncho-alveolar lavage, deep tracheal aspirates, and sputum) (48%), blood (12%), abscesses (15%), and urine (7%), and the remainder were from other sterile body fluids (CSF, bile, pericardial samples, peritoneal samples, pleural fluids, and catheters)	Broth micro-dilution test, confirmed with E-test
Cicek et al., 2014 [57]	Türkiye	ICU and non-ICU settings	101 clinical *A. baumannii* strains	November 2011 and July 2012	Single center	Yes	CLSI	0/101 (0.0%)	Respiratory specimens (tracheal aspirates 17%, sputum 36%), wounds (21.9%), blood (19%), catheters (3%), an abdominal specimen (0.9%), cerebrospinal fluid (0.9%), pleural fluid (0.9%) and urine (0.9%)	Vitek 2, confirmed with E-test
Direkel et al., 2015 [58]	Türkiye	ICU and non-ICU settings	531 hospitalized patients; colistin susceptibility assessed in a subset of 354 isolates from hospitalized patients of all ages	January 2012 to June 2014	Single center	Yes	CLSI	3/354 (0.8%)	Tracheal aspirates: 45%; urine: 17.3%; blood: 14.7%; wounds: 10. 4%; sputum: 7.1%; catheters: 5.5%	E-test (MIC determination)
Durdu et al., 2018 [59]	Türkiye	ICU	Adult ICU patients with nosocomial infection	January 2012 to November 2015	Five ICUs in a single hospital	NA	CLSI	1/350 (0.3%)	Respiratory samples: 258 (50.1%), blood: 68 (38.4%), urine: 15 (24.6%), others: 9 (33.3%)	VITEK 2
Erdem et al., 2022 [60]	Türkiye	ICU and non-ICU settings	Hospitalized patients with laboratory-confirmed HAI (123 *A. baumannii* isolates)	Between the years 2015 and 2019	Single center	NA	NA	3/123 (2.4%)	Respiratory specimens, blood, urine	Kirby–Bauer disk diffusion + VITEK 2 system
Ergin et al., 2013 [61]	Türkiye	Hospitalized patients at university Hospital (bloodstream infections)	Patients with BSI (100 *A. baumannii* isolates)	Between 2004 and 2010	Single center	Yes	CLSI	2/100 (2%)	Blood only	E-test
Ergonul et al., 2016 [62]	Türkiye	Hospitalized patients in the ICU	All patients with healthcare-associated Gram-negative bloodstream infections	1 January 2013 and 31 December 2013	Multi-center (17 tertiary hospitals)	Yes	CLSI	15/256 (6%)	Blood	Disk diffusion
Oncul et al., 2014 [63]	Türkiye	Burn ICU	Hospitalized burn patients with nosocomial infections	January 2001 and January 2012	Single center	Yes	CLSI	0/186 (0.0%)	Burn wounds, blood, respiratory samples, urine, and mixed infections	Kirby–Bauer disk diffusion
Öner et al., 2024 [64]	Türkiye	ICU (48.4%) and non-ICU settings (51.6%)	Inpatient and outpatient isolates (188 *A. baumannii* isolates)	Between 5 February 2020 and 3 March 2023	Multi-center	NA	EUCAST	20/188 (10.6%)	Blood: 54 (28.7%); sputum: 42 (22.3%); wounds: 35 (18.3%); other: 57 (30.6%)	Liquid micro-dilution method
Ülkü Tüzemen et al., 2022 [65]	Türkiye	Hospital wards and ICU (tertiary university hospital)	Hospitalized adult patients with bloodstream infection	January 2008 to December 2019	Single center	No	CLSI and EUCAST	16/864 (1.8%)	Blood	Kirby–Bauer disk diffusion
Uzunoglu et al., 2017 [66]	Türkiye	ICU (81.48%) and non-ICU settings	135 *A. baumannii* isolates from hospitalized patients of all ages	January Between June 2013 and September 2014	Single center	Yes	CLSI	0/135 (0%)	Respiratory specimens (62 tracheal aspirates (45.92%), 23 sputum (17.03%), 4 broncho-alveolar lavage (2.96%)) followed by 24 blood (17.77%), 12 urine (8.88%), 4 wounds (2.96%), 2 catheter tips (0.74%), 1 cerebrospinal fluid (0.74%) (n = 1), 1 pleural fluid (0.74%), 1 abscess (0.74%) and 1 tissue culture (0.74%)	Becton Dickinson (BD) Phoenix Automated Microbiology System
Yardimci and Arman, 2022 [67]	Türkiye	ICU and non-ICU settings, tertiary care hospital	Patients with BSI	Between January 2016 and December 2020	Single center	Yes	EUCAST	3/178 (1.7%)	Blood only	VITEK 2 compact system
Alsultan et al., 2013 [68]	Saudi Arabia	ICU patients	196 CRAB isolates	2008 and 2011	Multi-center	NA	BSAC	2/196 (1.0%)	Blood, respiratory samples, urine and wounds	Plate doubling dilution
Aly et al., 2016 [69]	Saudi Arabia	Patients of all ages	130 randomly selected *A. baumannii* clinical isolates	2006–2014.	Single center	Yes	CLSI	2/130 (1.5%)	Respiratory samples: 60 (46.2%), wounds and burns: 31 (23.8%), blood: 18 (13.8%), urine: 5 (3.8%), and others: 16 (12.3%)	MicroScan^®^ Walkaway system with E-test^®^ confirmation
Aly et al., 2014 [70]	Saudi Arabia	Patients of all ages	253 isolates	2006 and 2008	Single center	Yes	CLSI	3/253 (1.19%)	Respiratory samples: 117/253 (46.24%), wounds and burns: 61/253 (24.11%), blood: 34/253 (13.43%), urine: 8/253 (3.16%) and others: 33/253 (13.04%)	E-test
Badger-Emeka et al., 2024 [71]	Saudi Arabia	Archived *A. baumannii* isolates	177 170 randomly selected *A. baumannii* isolates	Over the periods of 2013–2014, 2016–2018, and 2019–2020	Single center	NA	CLSI/EUCAST	18%17%	Most isolates were from trans-tracheal aspirates (35.02%) and wound swabs (30%), followed by sputum (12.3%), blood (2.82%), bronchial alveolar lavage (1.69%), central venous catheters (1.69%), cerebrospinal fluid (1.23%), nasopharyngeal aspirates (1.69%), urine (3.4%), tissue culture (1.69%), incision drainage fluid (3.95%), and unspecified sources (4.52%)	Vitek 2
Elabd et al., 2015 [72]	Saudi Arabia	Hospitalized patients in all units (ICU: 61.1%)	Hospitalized patients with nosocomial infection (≥48 h admission)	October 2013 to March 2014	Single center	Yes	CLSI	5/108 (4.6%)	Tracheal aspirates: 31.5%, sputum: 29.6%, wound swabs: 22.2%, urine: 11.1%, blood: 5.6%, others: 5.6%	MicroScan Walkaway automated systems, and E-test
Baadani et al., 2013 [73]	Saudi Arabia	All units (ICU and non-ICU settings)	1307 clinical isolates of *A. baumannii*	January 2010 and December 2011	Bi-centric	Yes	CLSI	Overall: 23/1307 (1.8%)	Respiratory samples (33.0%), wounds (22.5%), blood (18.4%), urine (12.5%), others (13.7%)	VITEK 2 and Microscan automated system
Khaled et al., 2021 [74]	Saudi Arabia	Outpatient and inpatient settings	Patient of all ages hospitalized or treated outpatients from whom *A. baumannii* was isolated	1 January 2020 to 29 December 2020	Single center	NA	NA	166/342 (48.5%)	Respiratory samples: 50.6%, blood: 14%, wounds: 10.2%, urine: 9.6%, rectal samples: 9.4%, CSF, tissue, pleural fluid, trophic ulcers, sacral tissue, peritoneal fluid, intra-abdominal tissue, bedsores	Automated Phoenix system
Hafiz et al., 2023 [75]	Saudi Arabia	ICU	Adults critically ill patients admitted to the ICU (115 intubated patients)	March 2020 and March 2022	Single center	Yes	CLSI	15%	Respiratory specimens only (endotracheal aspirates and sputum)	Kirby–Bauer method or the BD Phoenix system
Saeed et al., 2010 [76]	Saudi Arabia	ICU	Hospitalized ICU patients	January to December 2009	Single center	Yes	CLSI	0/133 (0%)	Respiratory samples (38.8%), blood (33%), wounds/tissue (19.4%), CSF (1.2%), urinary tract (4.6%), and nose (2.9%)	Disk diffusion method
Shah et al., 2019 [77]	Saudi Arabia	Tertiary care hospital (university hospital) ICU and non-ICU settings	Patients with HAIs and CAIs (135 *A. baumannii* isolates)	2015–2016	Single center	Yes	CLSI	0/135 (0%)	Tracheal aspirates: 21.5%, blood: 20.7%, wound: 14.1%, urine: 14.1%, catheter: 6.7%, others: 23.0%	VITEK 2 (colistin reconfirmed by broth micro-dilution)
Alharbi et al., 2025 [78]	Saudi Arabia	Inpatients (96%) and outpatients	Hospitalized and outpatients of all ages (5777 *A. baumannii* isolates)	January 2013 to December 2023	Single center	NA	CLSI	Varied over time; 3.7% in 2023	Sputum, blood, urine, wounds and catheter tips	NA
AlFaris et al., 2024 [79]	Jordan	Clinical isolates	162 *A. baumannii* isolates	May 2018 to May 2019	Multi-center	NA	CLSI	2/162 (1.23%)	Sputum, blood, pus, urine, swabs, CSF, broncho-alveolar lavage, abdominal/pleural fluids, cancer tissue	E-test
Almomani et al., 2015 [80]	Jordan	Patients who were ≥16 years of age, admitted to a CCU, and diagnosed with VAP caused mainly by *A. baumannii*	121 *A. baumannii* from VAP cases	1 January 2007, and 30 June 2013	Single center	Yes	CLSI	0/121 (0.0%)	Bronchial wash, sputum, and pleural fluid	Kirby–Bauer disk diffusion method
Al-Tamimi et al., 2022 [81]	Jordan	Inpatient and outpatients of all ages	622 *A. baumannii* isolates	January 2010 to June 2020	Multi-center	Yes	CLSI	14/622 (2.3%)	Urine, respiratory samples (sputum and broncho-alveolar lavage), blood, wound pus, body fluids, and tissue	Vitek 2
Batarseh et al., 2015 [82]	Jordan	Hospital-wide data (isolates were collected from various clinical specimens across the hospital)	116 positive clinical samples	January to December 2013	Single center	Yes	CLSI	Total: 2/116 (1.7%), swabs: 1/57 (1.8%), blood: 1/24 (4.2%), sputum: 0/28 (0.0%), urine: 0/7 (0.0%)	Blood (n = 24), sputum (n = 28), urine (n = 7) and pus swabs (n = 57 from wounds, tips of catheters and body parts)	VITEK 2 automated system
Gharaibeh et al., 2024 [83]	Jordan	Outpatients and inpatients	150 *A. baumannii*; isolates were recovered from various clinical specimens collected	June 2021 and May 2022	Single center	Yes	CLSI	31/150 (20.6%)	Sputum (n = 80, 53.3%) and blood (n = 24, 16%), followed by urine (n = 15, 10%), wounds (n = 15, 10%), tips of central lines (n = 9, 6%), cerebrospinal fluid (n = 4, 2.7%) and pus (n = 3, 2%)	Micro-dilution method
Salloum et al., 2020 [84]	Lebanon	All hospital units	All patients with positive bacterial culture	January 2017 to June 2017	Bi-centric	Yes	NA	0/110 (0.0%)	NA	Disk diffusion
Itani et al., 2023 [85]	Lebanon	ICU (77%) and non-ICU settings in a [university tertiary care hospital]	Hospitalized adult patients	January 2021 to December 2021 (12-month period)	Single center	Yes	CLSI	0/111 (0.0%)	Deep tracheal aspirates (57, 51.4%), sputum (27, 24.3%), skin wounds/exudates (15, 13.5%), blood (11, 9.9%), and urine (1, 0.9%)	Kirby–Bauer disk diffusion method
Seleim et al., 2022 [86]	Egypt	ICU (65%) and non-ICU settings (university hospital clinical laboratories)	Hospitalized patients with *A. baumannii* infections	January 2020 to March 2021	Single center (multiple labs within university were used, part of the same hospital system)	NA	EUCAST	49/100 (49%)	Purulent discharge (32), sputum (29), blood (20), urine (14), pleural fluid (3), and ascitic fluid (2)	Broth micro-dilution
Mohammed et al., 2020 [87]	Egypt	ICU and non-ICU settings (mixed hospitalized population, including ICU-associated devices such as endotracheal tubes and catheters)	Hospitalized febrile patients with infection (all ages), 100 *A. baumannii* isolates	January 2014 and March 2019	Bi-centric	NA	CLSI	5/100 (5%)	Endotracheal tubes (ETTs): 29 (29%), blood: 12 (12%), sputum: 13 (13%), urine: 9 (9%), wounds: 6 (6%), central venous catheters (CVCs): 2 (2%), urinary tract catheters: 2 (2%), skin swabs: 2 (2%), throat swabs: 2 (2%)	Kirby–Bauer disk diffusion method
Kareem et al., 2020 [88]	Iraq	ICU (43%), internal medicine (29%), brain and neurosurgery (17%), infectious disease (10%) and others (2%)	Patient of all ages	2014–2018	Multi-center	NA	CLSI	32%	Urine (n = 120, 58%), blood (n = 50, 24%), cerebrospinal fluid (n = 25, 12%) and pus (n = 10, 5%)	Broth micro-dilution method
Jubair et al., 2025 [89]	Iraq	ICU (5.7%) and non-ICU settings	Patients attending the participating hospitals (175 *A. baumannii* isolates)	January 2023 and April 2024	Multi-center	Yes	CLSI	14/175 (8.0%)	Wound discharge (68, 38.8%), urine (51, 29%), blood (26, 14.8%), sputum (13, 7.4%), cerebrospinal fluid (CSF) (11, 6.2%), and broncho-alveolar lavage (BAL) (6, 3.4%)	Vitek-2 compact system
Alrahmany et al., 2021 [90]	Oman	Adult inpatients (>18 years)	282 isolates tested against colistin	1 January 2016 to 31 December 2017	Single center	Yes	CLSI	Blood: 0/22 (0.0%), respiratorysamples: 11/142 (7.7%), skin and soft tissue: 13/105 (12.4%), urine 0/42 (0.0%), others: 0/9 (0.0%) Overall resistance: 8.5% (24/282)	Blood, respiratory samples, skin and soft tissue, urine, and other body fluids/devices	NA
Sannathimmappa et al., 2021 [91]	Oman	Inpatient and outpatient settings	1890 *A. baumannii* isolates from inpatients and outpatients of all ages	January 2015 and December 2019	Single center	Yes	CLSI	Less than 1%	Skin and soft tissue specimens (551; 29.2%), respiratory secretions (519; 28%), urine (481; 25%), blood (160; 9%), catheter tips (48; 3%), and others (131; 7%)	Kirby–Bauer disc diffusion method
Vali et al., 2015 [92]	Kuwait	All units	Hospitalized patients (100 *A. baumannii* isolates)	2011 until 2012	Single center	NA	CLSI	3/100 (3%)	Urine (33%), wounds (18%)	Broth micro-dilution
Abukhalil et al., 2024 [93]	Palestine	All units (all inpatient records)	135 *A. baumannii* isolates from inpatients aged 18 years and above	1 November 2021, to 30 November 2022	Single center	NA	NA	1/135 (0.7%)	Blood, fluid, sputum, urine, and wounds	NA
Al Samawi et al., 2016 [94]	Qatar	Inpatients	239 patients with *Acinetobacter* infections	1 January 2012, to 31 December 2013	Single center	NA	CLSI	2/137 (1.4%)	Blood, respiratory tract samples, urine, wounds, tissue, CSF, synovial fluid, peritoneal fluid, abdominal drainage fluid	Broth micro-dilution
Sonnevend et al., 2013 [95]	United Arab Emirates	ICU (30%) and non-ICU settings	110 clinical *A. baumannii* isolates from hospitalized patients	Between March and November 2008	Multi-center	Yes	CLSI	1/110 (0.9%)	Respiratory samples (35.5%), wounds/soft tissue (30.0%), blood/catheters (14.5%), urine (11.8%), others (8.2%)	E-test

CLSI: Clinical & Laboratory Standards Institute, EUCAST: European Committee on Antimicrobial Susceptibility Testing, NA: not available, HAI: hospital-acquired infection, BSI: bloodstream infection, VAP: ventilator-associated pneumonia, ICU: intensive care unit, CRAB: carbapenem-resistant *A. baumannii*, CSAB: carbapenem-sensitive *A. baumannii*, CSF: cerebrospinal fluid, BAL: bronchoalveolar lavageronchoalveolar lavage, ETT: endotracheal tube, IV: intravenous.

## Data Availability

Data is contained within the article.

## References

[1] Lynch J.P., Zhanel G.G., Clark N.M. (2017). Infections due to *Acinetobacter baumannii* in the ICU: Treatment options. Semin. Respir. Crit. Care Med..

[2] Antimicrobial Resistance Collaborators (2022). Global burden of bacterial antimicrobial resistance in 2019: A systematic analysis. Lancet.

[3] Antunes L.C., Visca P., Towner K.J. (2014). *Acinetobacter baumannii*: Evolution of a global pathogen. Pathog. Dis..

[4] Poirel L., Jayol A., Nordmann P. (2017). Polymyxins: Antibacterial activity, susceptibility testing, and resistance mechanisms encoded by plasmids or chromosomes. Clin. Microbiol. Rev..

[5] Hussein N.H., Al-Kadmy I.M.S., Taha B.M., Hussein J.D. (2021). Mobilized colistin resistance (mcr) genes from 1 to 10: A comprehensive review. Mol. Biol. Rep..

[6] Al Atrouni A., Hamze M., Jisr T., Lemarié C., Eveillard M., Joly-Guillou M.L., Kempf M. (2016). Widespread of OXA-23-producing carbapenem-resistant *Acinetobacter baumannii* belonging to clonal complex II in different hospitals in Lebanon. Int. J. Infect. Dis..

[7] Alghanem S.S., Alfouzan W.A., Soliman M.M., Alroomi E., Alajmi S., Mahmoud T., Yagan J. (2026). Colistin use and outcomes in Kuwait public hospitals. Front. Drug Saf. Regul..

[8] Sehmen E., Yılmaz E.M., Pusa S.Y., Özdemir M., Yiğit Y. (2025). Exploring the evolution of *Acinetobacter baumannii* and *Pseudomonas aeruginosa* resistance during the COVID-19 era. Antimicrob. Steward. Healthc. Epidemiol..

[9] Abbasi E., Goudarzi H., Hashemi A., Chirani A.S., Ardebili A., Goudarzi M., Sharahi J.Y., Davoudabadi S., Talebi G., Bostanghadiri N. (2021). Decreased carO gene expression and OXA-type carbapenemases among extensively drug-resistant *Acinetobacter baumannii* strains isolated from burn patients in Tehran, Iran. Acta Microbiol. Immunol. Hung..

[10] Adibhesami H., Douraghi M., Zeraati H., Bazmi F., Rahbar M., Pourmand M.R., Tabrizi M.S., Aliramezani A., Ghourchian S. (2016). Carbapenem-resistant *Acinetobacter baumannii* (CRAB) recovered from burn patients. J. Pharm. Pharm. Sci..

[11] Aliakbarzade K., Farajnia S., Nik A.K., Zarei F., Tanomand A. (2014). Prevalence of aminoglycoside resistance genes in *Acinetobacter baumannii* isolates. Jundishapur J. Microbiol..

[12] Golafshani F.B., Kaboosi H., Armaki M.T., Ghadikolaii F.P., Fattahi E. (2019). Molecular investigation of integron types and imipenem-resistance encoded genes in *Acinetobacter baumannii* strains isolated from burns patients in Iran. Gene Rep..

[13] Bahador A., Farshadzadeh Z., Raoofian R., Mokhtaran M., Pourakbari B., Pourhajibagher M., Hashemi F.B. (2018). Association of virulence gene expression with colistin-resistance in *Acinetobacter baumannii*: Analysis of genotype, antimicrobial susceptibility, and biofilm formation. Ann. Clin. Microbiol. Antimicrob..

[14] Farajnia S., Azhari F., Alikhani M.Y., Hosseini M.K., Peymani A., Sohrabi N. (2013). Prevalence of PER and VEB type extended spectrum betalactamases among multidrug resistant *Acinetobacter baumannii* isolates in North-West of Iran. Iran. J. Basic Med. Sci..

[15] Farajnia S., Lotfi F., Dehnad A., Shojaie M., Raisi R., Rahbarnia L., Bazmani A., Naghili B., Shiry S. (2022). The molecular characterization of colistin-resistant isolates of *Acinetobacter baumannii* from patients at intensive care units. Iran. J. Microbiol..

[16] Farshadzadeh Z., Khoshnood S., Akrami S., Abbasi Montazeri E., Moradi M., Masihzadeh S., Daneshfar S., Navidifar T. (2025). Relationship between antibiotic resistance and biofilm-forming capacity with RND efflux pumps expression in clinical *Acinetobacter baumannii* isolates. Arch. Microbiol..

[17] Firoozeh F., Nikibakhsh M., Badmasti F., Zibaei M., Nikbin V.S. (2023). Clonal relatedness of carbapenem-resistant *Acinetobacter baumannii*: High prevalence of ST136pas in a burn center. Ann. Clin. Microbiol. Antimicrob..

[18] Ghahraman M.R., Hosseini-Nave H., Azizi O., Shakibaie M.R., Mollaie H.R., Shakibaie S. (2020). Molecular characterization of lpxACD and pmrA/B two-component regulatory system in the colistin resistance *Acinetobacter baumannii* clinical isolates. Gene Rep..

[19] Gholami M., Moshiri M., Ahanjan M., Salimi Chirani A., Hasannejad Bibalan M., Asadi A., Eshaghi M., Pournajaf A., Abbasian S., Kouhsari E. (2018). The diversity of class B and class D carbapenemases in clinical *Acinetobacter baumannii* isolates. Infez. Med..

[20] Goudarzi M., Azimi H. (2017). Dissemination of classes 1, 2, and 3 integrons in *Acinetobacter baumannii* strains recovered from intensive care units using polymerase chain reaction-restriction fragment length polymorphism. Jundishapur J. Microbiol..

[21] Hashemi B., Afkhami H., Khaledi M., Kiani M., Bialvaei A.Z., Fathi J., Sarley H., Divsalar S., Ahanjan M. (2020). Frequency of Metalo beta Lactamase genes, bla IMP1, INT 1 in *Acinetobacter baumanii* isolated from burn patients North of Iran. Gene Rep..

[22] Heidary M., Salimi Chirani A., Khoshnood S., Eslami G., Atyabi S.M., Nazem H., Fazilati M., Hashemi A., Soleimani S. (2017). Molecular detection of aminoglycoside-modifying enzyme genes in *Acinetobacter baumannii* clinical isolates. Acta Microbiol. Immunol. Hung..

[23] Izadi B., Souzani R., Farahani A., Mehrabian S., Mohajeri P. (2017). Frequency of class 2 integrons in multidrug-resistant *Acinetobacter baumannii* isolated from patients in West of Iran. Ann. Trop. Med. Public Health.

[24] Jasemi S., Douraghi M., Adibhesami H., Zeraati H., Rahbar M., Boroumand M.A., Aliramezani A., Ghourchian S., Mohammadzadeh M. (2016). Trend of extensively drug-resistant *Acinetobacter baumannii* and the remaining therapeutic options: A multicenter study in Tehran, Iran over a 3-year period. Lett. Appl. Microbiol..

[25] Karami F., Nazari R., Adeli H. (2021). Detection of Genes Encoding Metallo-beta-lactamases in Carbapenem Resistant *Acinetobacter baumannii*. J. Kerman Univ. Med. Sci..

[26] Khoshnood S., Eslami G., Hashemi A., Bahramian A., Heidary M., Yousefi N., Mohammdi F., Gholami M. (2017). Distribution of Aminoglycoside resistance genes among *Acinetobacter baumannii* strains isolated from burn patients in Tehran, Iran. Arch. Pediatr. Infect. Dis..

[27] Kooti S., Motamedifar M., Sarvari J. (2015). Antibiotic resistance profile and distribution of oxacillinase genes among clinical isolates of *Acinetobacter baumannii* in Shiraz teaching hospitals, 2012–2013. Jundishapur J. Microbiol..

[28] Meshkat Z., Salimizand H., Amini Y., Mansury D., Zomorodi A.R., Avestan Z., Jamee A., Falahi J., Farsiani H., Mojahed A. (2021). Detection of efflux pump genes in multi-resistant *Acinetobacter baumannii* ST2 in Iran. Acta Microbiol. Immunol. Hung..

[29] Mohajeri P., Farahani A., Feizabadi M.M., Ketabi H., Abiri R., Najafi F. (2013). Antimicrobial susceptibility profiling and genomic diversity of *Acinetobacter baumannii* isolates: A study in western Iran. Iran. J. Microbiol..

[30] Mohammadi F., Goudarzi H., Hashemi A., Nojookambari N.Y., Khoshnood S., Sabzehali F. (2017). Detection of ISAba1 in *Acinetobacter baumannii* strains carrying OXA genes isolated from iranian burns patients. Arch. Pediatr. Infect. Dis..

[31] Mohammadi M., Soroush S., Delfani S., Pakzad I., Abbaszadeh A., Bahmani M., Bogdanovic L., Taherikalani M. (2017). Distribution of class D carbapenemase and extended-spectrum β-lactamase genes among *Acinetobacter baumannii* isolated from burn wound and ventilator associated pneumonia infections. J. Clin. Diagn. Res. JCDR.

[32] Moosavian M., Ahmadi K., Shoja S., Mardaneh J., Shahi F., Afzali M. (2020). Antimicrobial resistance patterns and their encoding genes among clinical isolates of *Acinetobacter baumannii* in Ahvaz, Southwest Iran. MethodsX.

[33] Moosavian M., Sirous M., Shams N. (2017). Phenotypic and genotypic detection of extended spectrum β-lactamase and carbapenemases production including bla TEM, bla PER and bla NDM-1 genes among *Acinetobacter baumannii* clinical isolates. Jundishapur J. Microbiol..

[34] Nikibakhsh M., Firoozeh F., Badmasti F., Kabir K., Zibaei M. (2021). Molecular study of metallo-β-lactamases and integrons in *Acinetobacter baumannii* isolates from burn patients. BMC Infect. Dis..

[35] Owlia P., Azimi L., Gholami A., Asgari B., Larry A.R. (2012). ESBL- and MBL-mediated resistance in *Acinetobacter baumannii*: A global threat to burn patients. Infez. Med..

[36] Pourhajibagher M., Mokhtaran M., Esmaeili D., Bahador A. (2016). Antibiotic resistance patterns among *Acinetobacter baumannii* strains isolated from burned patients. Pharm. Lett..

[37] Rahimzadeh G., Valadan R., Rezai S., Khosravi M., Larijani L.V., Sheidaei S., Hevelaee E.N., Movahedi F.S., Rezai R., Rezai M.S. (2024). Evaluation of antibiotic resistance changes in *Acinetobacter baumannii* in the era of COVID-19 in Northern Iran. Iran. J. Microbiol..

[38] Ranaei M.A., Shahraki-Zahedan S., Mohagheghi-Fard A.H., Salimizand H., Ordoni R., Amini Y. (2020). Prevalence of the blaCTX-M and antibiotic resistance pattern among clinical isolates of *Acinetobacter baumannii* isolated from Zahedan, Southeast Iran. Gene Rep..

[39] Ranjbar R., Farahani A. (2019). Study of genetic diversity, biofilm formation, and detection of Carbapenemase, MBL, ESBL, and tetracycline resistance genes in multidrug-resistant *Acinetobacter baumannii* isolated from burn wound infections in Iran. Antimicrob. Resist. Infect. Control.

[40] Rostamani M., Bakht M., Rahimi S., Alizadeh S.A., Anari R.K., Khakpour M., Javadi A., Fardsanei F., Nikkhahi F. (2024). Phenotypic and genotypic determination of resistance to common disinfectants among strains of *Acinetobacter baumannii* producing and non-producing biofilm isolated from Iran. BMC Microbiol..

[41] Safari M., Saidijam M., Bahador A., Jafari R., Alikhani M.Y. (2013). High prevalence of multidrug resistance and metallo-beta-lactamase (MbetaL) producing *Acinetobacter baumannii* isolated from patients in ICU wards, Hamadan. Iran. J. Res. Health Sci..

[42] Salehi B., Goudarzi H., Nikmanesh B., Houri H., Alavi-Moghaddam M., Ghalavand Z. (2018). Emergence and characterization of nosocomial multidrug-resistant and extensively drug-resistant *Acinetobacter baumannii* isolates in Tehran, Iran. J. Infect. Chemother..

[43] Salehi B., Ghalavand Z., Mohammadzadeh M., Maleki D.T., Kodori M., Kadkhoda H. (2019). Clonal relatedness and resistance characteristics of OXA-24 and -58 producing carbapenem-resistant *Acinetobacter baumannii* isolates in Tehran, Iran. J. Appl. Microbiol..

[44] Sedaghat A., Khadem-Rezaiyan M., Ahmadabadi A., Abbaspour H., Youssefi M., Shirzad M.M., Esfahani M., Mirzaei M., Ramezani M. (2019). Antibacterial resistance pattern of *Acinetobacter baumannii* in burn patients in Northeast of Iran. Jundishapur J. Microbiol..

[45] Sepahvand S., Doudi M., Davarpanah M.A., Bahador A., Ahmadi M. (2016). Analyzing pmrA and pmrB genes in *Acinetobacter baumannii* resistant to colistin in Shahid Rajai Shiraz, Iran Hospital by PCR: First report in Iran. Pak. J. Pharm. Sci..

[46] Shoja S., Moosavian M., Peymani A., Tabatabaiefar M.A., Rostami S., Ebrahimi N. (2013). Genotyping of carbapenem resistant *Acinetobacter baumannii* isolated from tracheal tube discharge of hospitalized patients in intensive care units, Ahvaz, Iran. Iran. J. Microbiol..

[47] Sohrabi N., Farajnia S., Akhi M.T., Nahaei M.R., Naghili B., Peymani A., Amiri Z., Rezaee M.A., Saeedi N. (2012). Prevalence of OXA-type β-lactamases among *Acinetobacter baumannii* isolates from Northwest of Iran. Microb. Drug Resist..

[48] Tayebi Z., Doust R.H., Rahimi M.K., Siadat S.D., Goudarzi M. (2019). Distribution of different carbapenemase genes in carbapenem-resistant *Acinetobacter baumannii* strains isolated from intensive care: A two year multi-center study in Tehran, Iran. Gene Rep..

[49] Vahhabi A., Hasani A., Rezaee M.A., Baradaran B., Hasani A., Kafil H.S., Soltani E. (2021). Carbapenem resistance in *Acinetobacter baumannii* clinical isolates from northwest Iran: High prevalence of OXA genes in sync. Iran. J. Microbiol..

[50] Zarifi E., Eslami G., Khaledi A., Vakili M., Vazini H., Zandi H. (2017). Prevalence of ESBLs in *Acinetobacter baumannii* isolated from intensive care unit (ICU) of Ghaem hospital, Mashhad, Iran. J. Pure Appl. Microbiol..

[51] Zendegani E., Dolatabadi S. (2020). The efficacy of imipenem conjugated with synthesized silver nanoparticles against *Acinetobacter baumannii* clinical isolates, Iran. Biol. Trace Elem. Res..

[52] Zafari M., Feizabadi M.M., Jafari S., Abdollahi A., Sabokbar A. (2017). High prevalence of OXA-type carbapenemases among *Acinetobacter baumannii* strains in a teaching hospital of Tehran. Acta Microbiol. Immunol. Hung..

[53] Akıllı F.M., İlki A. (2025). Evaluation of Colistin Susceptibility in Carbapenem-Resistant *Acinetobacter baumannii* Isolates Using Broth Microdilution, MICRONAUT-MIC-Strip, and VITEK MS. MicrobiologyOpen.

[54] Aydın M., Ergönül Ö., Azap A., Bilgin H., Aydın G., Çavuş S.A., Demiroğlu Y.Z., Alışkan H.E., Memikoğlu O., Menekşe Ş. (2018). Rapid emergence of colistin resistance and its impact on fatality among healthcare-associated infections. J. Hosp. Infect..

[55] Çetin B., Tekin S., Güneş T. (2022). Evaluation of *Acinetobacter baumannii* Infections and Skin Colonization in the Neonatal Intensive Care Unit Yenidoğan Yoğun Bakım Ünitesinde *Acinetobacter baumannii* Enfeksiyonları ve Cilt Kolonizasyonunun Değerlendirilmesi. Cocuk Enfeksiyon Derg..

[56] Çağlan E., Nigiz Ş., Sancak B., Gür D. (2020). Resistance and heteroresistance to colistin among clinical isolates of *Acinetobacter baumannii*. Acta Microbiol. Immunol. Hung..

[57] Cicek A.C., Saral A., Iraz M., Ceylan A., Duzgun A.O., Peleg A.Y., Sandalli C.E. (2014). OXA-and GES-type β-lactamases predominate in extensively drug-resistant *Acinetobacter baumannii* isolates from a Turkish University Hospital. Clin. Microbiol. Infect..

[58] Direkel Ş., Uzunoğlu E., Keleş S., Yapar K. (2015). Antibiotic Resistance Rates of *Acinetobacter Baumannii* Strains Isolated from Various Clinical Samples in Giresun Prof. Dr. Atilla ilhan Özdemir State Hospital. Gazi Med. J..

[59] Durdu B., Kritsotakis E.I., Lee A.C., Torun P., Hakyemez I.N., Gultepe B., Aslan T. (2018). Temporal trends and patterns in antimicrobial-resistant Gram-negative bacteria implicated in intensive care unit-acquired infections: A cohort-based surveillance study in Istanbul, Turkey. J. Glob. Antimicrob. Resist..

[60] Erdem I., Yıldırım I., Safak B., Karaali R., Erdal B., Ardic E., Dogan M., Kardan M.E., Kavak C., Sahin Karadil K. (2022). A 5-year surveillance of healthcare-associated infections in a university hospital: A retrospective analysis. SAGE Open Med..

[61] Ergin A., Hascelik G., Eser O.K. (2013). Molecular characterization of oxacillinases and genotyping of invasive *Acinetobacter baumannii* isolates using repetitive extragenic palindromic sequence-based polymerase chain reaction in Ankara between 2004 and 2010. Scand. J. Infect. Dis..

[62] Ergönül Ö., Aydin M., Azap A., Başaran S., Tekin S., Kaya Ş., Gülsün S., Yörük G., Kurşun E., Yeşilkaya A. (2016). Healthcare-associated Gram-negative bloodstream infections: Antibiotic resistance and predictors of mortality. J. Hosp. Infect..

[63] Öncül O., Öksüz S., Acar A., Ülkür E., Turhan V., Uygur F., Ulçay A., Erdem H., Özyurt M., Görenek L. (2014). Nosocomial infection characteristics in a burn intensive care unit: Analysis of an eleven-year active surveillance. Burns.

[64] Öner S.Z., Er H., Çalışkan A., Demir M., Şenol H., Kaleli İ. (2024). An evaluation of clonal relationship by PFGE method in *Acinetobacter baumannii* isolates, examining integron presence and antibiotic resistance. J. Infect. Dev. Ctries..

[65] Ülkü Tüzemen N., Payaslioğlu M., Özakin C., Ener B., Akalin H. (2022). Trends of Bloodstream Infections in a University Hospital During 12 Years. Pol. J. Microbiol..

[66] Uzunoglu E., Direkel S., Kocbiyik M., Uludag S.K., Cicek A.C. (2017). Co-existance of isaba1/blaoxa-51/23 is increasing in carbapenem rersistant *Acinetobacter baumannii* isolates in Turkey. Acta Medica Mediterr..

[67] Yardimci A.C., Arman D. (2022). Prevalence and antimicrobial resistance of bloodstream infections caused by ESKAPEEc pathogens: A five-year analysis. Jundishapur J. Microbiol..

[68] Alsultan A.A., Evans B.A., Elsayed E.A., Al-Thawadi S.I., Al-Taher A.Y., Amyes S.G., Al-Dughaym A.M., Hamouda A. (2013). High frequency of carbapenem-resistant *Acinetobacter baumannii* in patients with diabetes mellitus in Saudi Arabia. J. Med. Microbiol..

[69] Aly M.M., Abu Alsoud N.M., Elrobh M.S., Al Johani S.M., Balkhy H.H. (2016). High prevalence of the PER-1 gene among carbapenem-resistant *Acinetobacter baumannii* in Riyadh, Saudi Arabia. Eur. J. Clin. Microbiol. Infect. Dis..

[70] Aly M., Tayeb H.T., Al Johani S.M., Alyamani E.J., Aldughaishem F., Alabdulkarim I., Balkhy H.H. (2014). Genetic diversity of OXA-51-like genes among multidrug-resistant *Acinetobacter baumannii* in Riyadh, Saudi Arabia. Eur. J. Clin. Microbiol. Infect. Dis..

[71] Badger-Emeka L.I., Emeka P.M., Quadri S.A. (2024). Periodic phenotypic profile analysis of *Acinetobacter baumannii* drug resistance characteristics showing the emergence of PDR. Eur. Rev. Med. Pharmacol. Sci..

[72] Elabd F.M., Al-Ayed M.S., Asaad A.M., Alsareii S.A., Qureshi M.A., Musa H.A. (2015). Molecular characterization of oxacillinases among carbapenem-resistant *Acinetobacter baumannii* nosocomial isolates in a Saudi hospital. J. Infect. Public Health.

[73] Baadani A.M., Thawadi S.I., El-Khizzi N.A., Omrani A.S. (2013). Prevalence of colistin and tigecycline resistance in *Acinetobacter baumannii* clinical isolates from 2 hospitals in Riyadh Region over a 2-year period. Saudi Med. J..

[74] Khaled J.M., Alharbi N.S., Siddiqi M.Z., Alobaidi A.S., Nauman K., Alahmedi S., Almazyed A.O., Almosallam M.A., Al Jurayyan A.N. (2021). A synergic action of colistin, imipenem, and silver nanoparticles against pandrug-resistant *Acinetobacter baumannii* isolated from patients. J. Infect. Public Health.

[75] Hafiz T.A., Alghamdi S.S., Mubaraki M.A., Alghamdi S.S., Alothaybi A., Aldawood E., Alotaibi F. (2023). A two-year retrospective study of multidrug-resistant *Acinetobacter baumannii* respiratory infections in critically Ill patients: Clinical and microbiological findings. J. Infect. Public Health.

[76] Saeed N.K., Kambal A.M., El-Khizzi N.A. (2010). Antimicrobial-resistant bacteria in a general intensive care unit in Saudi Arabia. Saudi Med. J..

[77] Shah M.W., Yasir M., Farman M., Jiman-Fatani A.A., Almasaudi S.B., Alawi M., El-Hossary D., Azhar E.I. (2019). Antimicrobial susceptibility and molecular characterization of clinical strains of *Acinetobacter baumannii* in Western Saudi Arabia. Microb. Drug Resist..

[78] Alharbi O., Al-Said H.M., Ashgar S.S., Jalal N.A., Faidah H., Momenah A.M., Johargy A.K., Bantun F., Qashqari F.S., Faidah O.H. (2025). Prevalence and Antibiogram pattern of *Acinetobacter baumannii* from 2013 to 2023 in a tertiary Care Hospital in the Western Region of Saudi Arabia. Antibiotics.

[79] AlFaris E.A., Al-Karablieh N., Odat N.A., Rafei R. (2024). Carbapenem-resistant *Acinetobacter baumannii* from Jordan: Complicated carbapenemase combinations. Jordan J. Biol. Sci..

[80] Almomani B.A., McCullough A., Gharaibeh R., Samrah S., Mahasneh F. (2015). Incidence and predictors of 14-day mortality in multidrug-resistant *Acinetobacter baumannii* in ventilator-associated pneumonia. J. Infect. Dev. Ctries..

[81] Al-Tamimi M., Albalawi H., Alkhawaldeh M., Alazzam A., Ramadan H., Altalalwah M., Alma’aitah A., Al Balawi D.A., Shalabi S., Abu-Raideh J. (2022). Multidrug-resistant *Acinetobacter baumannii* in Jordan. Microorganisms.

[82] Batarseh A., Al-Sarhan A., Maayteh M., Al-Khatirei S., Alarmouti M. (2016). Antibiogram of multidrug resistant *Acinetobacter baumannii* isolated from clinical specimens at King Hussein Medical Centre, Jordan: A retrospective analysis. East Mediterr. Health J..

[83] Gharaibeh M.H., Abandeh Y.M., Elnasser Z.A., Lafi S.Q., Obeidat H.M., Khanfar M.A. (2024). Multi-drug resistant *Acinetobacter baumannii*: Phenotypic and genotypic resistance profiles and the associated risk factors in teaching hospital in Jordan. J. Infect. Public Health.

[84] Salloum S., Michel T.A., Tayyara L. (2020). Bacterial resistance to antibiotics and associated factors in two hospital centers in Lebanon from January 2017 to June 2017. Infect. Prev. Pract..

[85] Itani R., Khojah H.M., Karout S., Rahme D., Hammoud L., Awad R., Abu-Farha R., Mukattash T.L., Raychouni H., El-Lakany A. (2023). *Acinetobacter baumannii*: Assessing susceptibility patterns, management practices, and mortality predictors in a tertiary teaching hospital in Lebanon. Antimicrob. Resist. Infect. Control.

[86] Seleim S.M., Mostafa M.S., Ouda N.H., Shash R.Y. (2022). The role of pmrCAB genes in colistin-resistant *Acinetobacter baumannii*. Sci. Rep..

[87] Mohammed M.A., Ahmed M.T., Anwer B.E., Aboshanab K.M., Aboulwafa M.M. (2020). Propranolol, chlorpromazine and diclofenac restore susceptibility of extensively drug-resistant (XDR)-*Acinetobacter baumannii* to fluoroquinolones. PLoS ONE.

[88] Kareem S.M. (2020). Emergence of mcr-and fosA3-mediated colistin and fosfomycin resistance among carbapenem-resistant *Acinetobacter baumannii* in Iraq. Meta Gene.

[89] Jubair H.H., Mezher M.J., Mayea N.A. (2025). Molecular Epidemiology and MLST-Based Typing of Pandrug-Resistant *Acinetobacter baumannii* Clinical Isolates in Iraq: A Cross-Sectional Study. Iran. J. Pathol..

[90] Alrahmany D., Omar A.F., Harb G., El Nekidy W.S., Ghazi I.M. (2021). *Acinetobacter baumannii* infections in hospitalized patients, treatment outcomes. Antibiotics.

[91] Sannathimmappa M.B., Nambiar V., Aravindakshan R. (2021). Antibiotic resistance pattern of *Acinetobacter baumannii* strains: A retrospective study from Oman. Saudi J. Med. Med. Sci..

[92] Vali L., Dashti K., Opazo-Capurro A.F., Dashti A.A., Al Obaid K., Evans B.A. (2015). Diversity of multi-drug resistant *Acinetobacter baumannii* population in a major hospital in Kuwait. Front. Microbiol..

[93] Abukhalil A.D., Barakat S.A., Mansour A., Al-Shami N.M., Naseef H. (2024). ESKAPE pathogens: Antimicrobial resistance patterns, risk factors, and outcomes a retrospective cross-sectional study of hospitalized patients in Palestine. Infect. Drug Resist..

[94] Al Samawi M.S., Khan F.Y., Eldeeb Y., Almaslamani M., Alkhal A., Alsoub H., Ghadban W., Howady F., Hashim S. (2016). *Acinetobacter* infections among adult patients in Qatar: A 2-year hospital-based study. Can. J. Infect. Dis. Med. Microbiol..

[95] Sonnevend A., Ghazawi A., Al Munthari N., Pitout M., Hamadeh M.B., Hashmey R., Girgis S.K., Sheikh F.A., Al Haj M., Nagelkerke N. (2013). Characteristics of epidemic and sporadic strains of *Acinetobacter baumannii* isolated in Abu Dhabi hospitals. J. Med. Microbiol..

[96] Bostanghadiri N., Narimisa N., Mirshekar M., Dadgar-Zankbar L., Taki E., Navidifar T., Darban-Sarokhalil D. (2024). Prevalence of colistin resistance in clinical isolates of *Acinetobacter baumannii*: A systematic review and meta-analysis. Antimicrob. Resist. Infect. Control.

[97] Pormohammad A., Mehdinejadiani K., Gholizadeh P., Nasiri M.J., Mohtavinejad N., Dadashi M., Karimaei S., Safari H., Azimi T. (2020). Global prevalence of colistin resistance in clinical isolates of *Acinetobacter baumannii*: A systematic review and meta-analysis. Microb. Pathog..

[98] Wang Y., Sholeh M., Yang L., Shakourzadeh M.Z., Beig M., Azizian K. (2025). Global trends of ceftazidime–avibactam resistance in gram-negative bacteria: Systematic review and meta-analysis. Antimicrob. Resist. Infect. Control.

[99] Qureshi Z.A., Hittle L.E., O’hara J.A., Rivera J.I., Syed A., Shields R.K., Pasculle A.W., Ernst R.K., Doi Y. (2015). Colistin-resistant *Acinetobacter baumannii*: Beyond carbapenem resistance. Clin. Infect. Dis..

[100] Vrysis C., Kontogiannis D.S., Ntourakis D., Kakoullis S.A., Falagas M.E. (2025). Risk factors for colistin-resistant, extensively drug-resistant (XDR) and pandrug-resistant (PDR) *Acinetobacter baumannii* infections: A review. Expert Rev. Anti-Infect. Ther..

[101] Page M.J., Moher D., Bossuyt P.M., Boutron I., Hoffmann T.C., Mulrow C.D., Shamseer L., Tetzlaff J.M., Akl E.A., Brennan S.E. (2021). PRISMA 2020 explanation and elaboration: Updated guidance and exemplars for reporting systematic reviews. BMJ.

[102] Higgins J.P., Thompson S.G., Deeks J.J., Altman D.G. (2003). Measuring inconsistency in meta-analyses. BMJ.

